# Comprehensive Evolutionary Analysis of *CPP* Genes in *Brassica napus* L. and Its Two Diploid Progenitors Revealing the Potential Molecular Basis of Allopolyploid Adaptive Advantage Under Salt Stress

**DOI:** 10.3389/fpls.2022.873071

**Published:** 2022-04-25

**Authors:** Mengdi Li, Fan Wang, Jiayu Ma, Hengzhao Liu, Hang Ye, Peng Zhao, Jianbo Wang

**Affiliations:** ^1^Key Laboratory of Resource Biology and Biotechnology in Western China, Ministry of Education, College of Life Sciences, Northwest University, Xi’an, China; ^2^State Key Laboratory of Hybrid Rice, College of Life Sciences, Wuhan University, Wuhan, China

**Keywords:** adaptive advantage, *Brassica napus*, *CPP* gene family, gene expression, physiological response, salt stress, allopolyploidization

## Abstract

Allopolyploids exist widely in nature and have strong environmental adaptability. The typical allopolyploid *Brassica napus* L. is a widely cultivated crop, but whether it is superior to its diploid progenitors in abiotic stress resistance and the key genes that may be involved are not fully understood. *Cystein-rich polycomb-like protein* (*CPP*) genes encode critical transcription factors involved in the response of abiotic stress, including salt stress. To explore the potential molecular basis of allopolyploid adaptation to salt stress, we comprehensively analyzed the characteristics and salt stress response of the *CPP* genes in *B. napus* and its two diploid progenitors in this study. We found some molecular basis that might be associated with the adaptability of *B. napus*, including the expansion of the CPP gene family, the acquisition of introns by some *BnCPPs*, and abundant *cis*-acting elements upstream of *BnCPPs*. We found two duplication modes (whole genome duplication and transposed duplication) might be the main reasons for the expansion of CPP gene family in *B. napus* during allopolyploidization. *CPP* gene expression levels and several physiological indexes were changed in *B. napus* and its diploid progenitors after salt stress, suggesting that *CPP* genes might play important roles in the response of salt stress. We found that some *BnCPPs* might undergo new functionalization or subfunctionalization, and some *BnCPPs* also show biased expression, which might contribute to the adaptation of *B. napus* under saline environment. Compared with diploid progenitors, *B. napus* showed stronger physiological responses, and *BnCPP* gene expression also showed higher changes after salt stress, indicating that the allopolyploid *B. napus* had an adaptive advantage under salt stress. This study could provide evidence for the adaptability of polyploid and provide important clues for the study of the molecular mechanism of salt stress resistance in *B. napus*.

## Introduction

Allopolyploidization is a powerful driving force for plant speciation and species diversity (Abbott et al., 2013; Soltis et al., 2016). This process makes the plants undergo a series of genetic and epigenetic changes, which may lead to the formation of new phenotypes, thus promoting the successful establishment of new allopolyploids and enabling it to adapt to the diverse ecological environment (Adams and Wendel, 2005; Chen, 2007; Jackson and Chen, 2010; Madlung, 2013; Yoo and Wendel, 2014). Natural allotetraploid *Brassica napus* (2n = 4x = 38, A_n_A_n_C_n_C_n_) is one of the most widely cultivated oil crops, which was formed by hybridization and genome doubling of diploid *B. rapa* (2n = 20, A_r_) and *B. oleracea* (2n = 18, C_o_) about 7,500 years ago, and its domestication and cultivation history were relatively short (Chalhoub et al., 2014). Therefore, natural *B. napus* and its diploid progenitors, *B. rapa* and *B. oleracea*, are a group of ideal material for investigating allopolyploidization event (Prakash et al., 2009). As an allotetraploid, *B. napus* has advantages than the diploid *Brassica* species in many aspects such as environmental adaptability (Segraves, 2017). Studies have found that rapeseed can accumulate erucic acid through erucic acid metabolic pathways under salt stress, and high erucic acid genes are related to the stress resistant genes (Ashraf and McNeilly, 2004; Wang et al., 2020), which implied that rapeseed might have the ability to resistant the salt stress. However, the potential molecular basis of the adaptive advantages in allopolyploids was still largely unknown.

Cystein-rich polycomb-like protein (CPP) contains one or two cysteine-rich (CXC) domains and a short sequence R that connects two CXC domains (Riechmann et al., 2000; Andersen et al., 2007; Zhang et al., 2015). Many studies reported *CPP* genes showed response to a variety of abiotic stresses in plants. For example, the expression of 15 *CPP* genes was significantly increased after 24 h of drought treatment in soybean (Zhang et al., 2015). *CsCPP* genes engaged in rapid stress responses under abiotic stresses (such as low temperature, drought, salt and abscisic acid) in cucumber (Zhou et al., 2018). The expression of *ZmCPP* genes in maize changed under extreme temperature, salt and drought stresses (Song et al., 2016). Studies have also reported the specific function of *CPP* genes. *TSO1*, the first *CPP* gene identified in *Arabidopsis thaliana*, highly expressed in flowers, developing ovules and microspores of Arabidopsis, and the lack of *TSO1* affected the karyokinesis and cytokinesis of cells (Hauser et al., 1998, 2000; Song et al., 2000). Further studies by Wang et al. (2018) found that *TSO1* played a key role in regulating cell cycle and cell fate, and was involved in regulating the development of plant stems and roots. In soybean, transcription factor CPP1 was involved in the regulation of the symbiotic nodules development by regulating the expression of *GMLBC3* (Cvitanich et al., 2000). In addition, Yang et al. (2008) first identified 8 *CPP* genes in Arabidopsis and 11 *CPP* genes in rice, and they found that the CXC domain and the R sequence might have coevolved during evolution. With the release and improvement of genomic information of many species, more CPP gene families in different species have been studied. For example, 20, 13, and 5 *CPP* genes were identified in soybean, maize, cucumber and wheat, respectively (Zhang et al., 2015; Song et al., 2016; Zhou et al., 2018). In general, the CPP gene family has only been systematically studied in a few species, and most of them are diploid species. Considering that the comparative analysis of CPP gene family between allopolyploid and its diploid parents/progenitors can provide new insights into the molecular basis of environmental adaptation in allopolyploids, it is necessary to identify and analyze CPP gene families in allopolyploid plants.

In this study, we comprehensively analyzed the characteristics and salt stress response (including physiological and gene expression changes) of the CPP gene family in *B. napus* and its two diploid progenitors, *B. rapa* and *B. oleracea*. We also explored the evolutionary changes of the CPP gene family in the allotetraploid *B. napus* compared with its progenitors, and uncover the potential molecular basis of the advantage of salt stress resistance in allotetraploid *B. napus*. Our results provide important clues for researchers to explore the roles of *CPP* genes in salt stress tolerance, and will deepen our understanding of the evolution of the molecular basis for polyploid stress resistance.

## Materials and Methods

### Identification and Duplication Mode of *CPP* Genes

All genome data of *B. rapa*, *B. oleracea* and *B. napus* used in this study were obtained from *Brassica* BRAD database (Cheng et al., 2011). Three methods were used to obtain candidate CPP gene family members. First, 8 AtCPP proteins were used as query sequences (downloaded from TAIR database^1^) to perform Blastp alignment (*E*-value < 1e^––5^) with all protein sequences of three *Brassica* species. Second, the HMM file of the CXC domain (ID: PF03638) was downloaded from the Pfam database^2^, and then the software HMMER was used to search this domain in all protein sequences of three species (*E*-value < 1e^––5^). Third, the syntenic genes of 8 *AtCPP* were searched in three *Brassica* species using the syntenic gene search function of BRAD database. All the members obtained by these three methods (i.e., the union of the members) were candidate members of the CPP gene family. Furthermore, Smart database^3^, Pfam database and CDD database^4^ were used to detect the integrity of the CXC domain of all the candidate CPP proteins, and the candidate members with sequence deletion in the domain were removed to obtain the final CPP gene family members. The duplication modes of all *CPP* genes were analyzed by the online website PlantDGD^5^. CDS sequences of three *Brassica* species were downloaded from BRAD database, and the CDS sequences of *CPP* genes of each species were compared using Blastn program. If the consistency and coverage of two CDS were both greater than 80%, they were considered as segmental duplicated gene pairs (Zhou et al., 2004). The software DNAsp5 (Librado and Rozas, 2009) was used to estimate the non-synonymous (*K*a) and synonymous substitution (*K*s) values between these gene pairs, and then the selection pressure of *CPP* genes in *B. napus* and its progenitors was determined based on Ka/Ks value.

### Phylogenetic Tree Construction and Characterization Analysis of CPP Proteins

CPP proteins from five species (*B. rapa*, *B. oleracea*, *B. napus*, Arabidopsis and rice) were aligned using the Clustal X program in MEGA software (v7.0; Kumar et al., 2016). According to the alignment results, the MEGA software was used to perform phylogenetic analysis of these CPP proteins utilizing the neighbor-joining (NJ) method with 1000 bootstrap replicates. The phylogenetic tree was decorated by the online website Tree of Life (iTOL^6^). According to the information provided by Smart database, IBS software (v1.0.3) was used to show the distribution of CXC domain on CPP proteins. The software Clustal W was used to align the CXC domains of all CPP proteins from the three *Brassica* species. The online website MEME^7^ was used to predict the characteristic motifs of CPP proteins of three species, and the number and length of the predicted motifs were set as 10 and 6–50 aa, respectively. The structure of the *CPP* genes was analyzed using the online tool GSDS^8^.

### Chromosomal Localization and Synteny Analysis of *CPP* Genes

The location information of all identified *CPP* genes was obtained from BRAD database, and their positions on chromosomes were visualized using the MapInspector software^9, 10^. The syntenic *CPP* genes were searched by the syntenic gene search function of BRAD database, and the synteny relationship between *CPP* genes was showed by Circos software (Krzywinski et al., 2009).

### Physicochemical Properties and Subcellular Localization Prediction of CPP Proteins

Online website ExPASy^11^ was used to predicate the physicochemical properties of CPP proteins, including the number of amino acids, molecular weight (MW), isoelectric point (pI), the grand average of hydropathy (GRAVY) and instability index (II). Subcellular localization of CPP proteins in three *Brassica* species was predicted using the online tool WoLF PSORT^12^.

### The *Cis*-Acting Elements in the Promoter of *CPP* Genes and Their Expression Patterns

The 2000 bp upstream of the transcription start site of *CPP* genes were considered to be the promoter region of *CPP* genes. The *cis*-acting elements in the promoter region of *CPP* genes were predicted using website PlantCARE^13^. Gene expression data from four organs in three *Brassica* species were derived from our previous studies (Li et al., 2020), and the raw data of which were deposited in the NCBI database (accession number: SRR7816633-SRR7816668). The heatmap was created using the software HemI.

### Plant Materials and Treatments

The experimental materials used in this study were *B. napus* (cv. Darmor), *B. rapa* (cv. Chiifu) and *B. oleracea* (cv. Jinzaosheng). Seeds were placed on wet double filter paper in a culture dish for germination in the light incubator (temperature: 22°C, light/dark: 16 h/8 h). When cotyledons of the seedlings were fully unfolded, placed them in black pots in which an equal amount of vermiculite and vegetative soil were mixed. The seedlings then grown in a light incubator under the same conditions, during which 1/2 Hoagland’s nutrient solution were regularly applied. Seedlings growing for 4 weeks were subjected to salt stress. The materials in the experimental group were irrigated with NaCl solution with a concentration of 300 mM (once a day, 30 ml), and the materials in the control group were irrigated with the same amount of distilled water for 4 days. Take the second to fifth leaves of the seedlings and freeze them immediately in liquid nitrogen for further use. Each species/treatment has three biological replicates. The experimental materials used in this study were shown in the Supplementary Figure 1.

### Measurement of Physiological Indexes

Three enzyme activities, including peroxidase (POD), superoxide dismutase (SOD) and catalase (CAT), were quantified according to the method of Cao et al. (2017), with minor modifications as described by Xu et al. (2020). The contents of proline (Pro), soluble sugar, soluble protein, and chlorophyll (Chl a + Chl b) were measured according to Quinet et al. (2012).

### RNA Isolation and Quantitative Real-Time PCR (qRT-PCR) Analysis

Total RNAs from each sample was isolated using the Trizol reagent (Invitrogen, Carlsbad, CA, United States) according to the instructions. The quality of RNA samples was checked on 1% agarose gel. The first strand complementary DNA (cDNA) was synthesized using Moloney Murine Leukemia Virus Reverse Transcriptase (M-MLV RT, Promega, Madison, WI, United States) following the manufacturer’s instructions. Then, cDNA was diluted 10-fold as the templates of qRT-PCR. The qRT-PCR reactions were performed using the ABI Step One Plus Real-Time PCR System (Applied Biosystems, Carlsbad, CA, United States) with the SYBR kit. The program was set to 94°C for 5 min; 38 cycles of 94°C for 30 s, 55°C for 30 s and 72°C for 20 s; and 72°C for 10 min. Three biological and three technical repeats were used for each sample. The *ACT2/7* gene was used as internal control to standardize the results and the relative expression level of selected genes was calculated using the delta-delta threshold cycle (2^–ΔΔCt^) method. Gene-specific primers for qRT-PCR were designed by Primer 5 software and the primer sequences were listed in Supplementary Table 1.

## Results

### *CPP* Gene Family Expanded in Allotetraploid *Brassica napus*

To ensure that CPP gene families in *B. napus* and its two diploid progenitors are fully identified, we used three methods to obtain candidate members. The three methods include Blastp alignment, HMMER scanning, and syntenic gene searching (see section “Identification and duplication mode of CPP genes” in Materials and Methods section). A total of 18, 19, and 37 candidate *CPP* genes were initially obtained in *B. rapa*, *B. oleracea* and *B. napus*. The integrity of the CXC domain of all candidate proteins was examined, and which with complete domain were identified as the final CPP gene family members. Finally, 15, 10, and 34 members were identified in *B. rapa*, *B. oleracea*, and *B. napus* (Table 1). The identified members were named according to the previous studies (Li et al., 2019b; Wang et al., 2019; Table 1).

**TABLE 1:** The *CPP* gene family information in *B. napus* and its diploid progenitors.

Gene name	Gene ID	Chromosome	Gene position		Intron number	Orthologous gene in *Arabidopsis*
			**Start**	**End**		
*BrCPP1*	Bra041042	Scaffold000410	7858	9837	6	AT2G20110/*AtCPP*1
*BrCPP2*	Bra001128	A03	14899606	14901982	7	AT3G04850/*AtCPP*2
*BrCPP3*	Bra021165	A01	22801607	22803170	5	AT3G16160/*AtCPP*3
*BrCPP4a*	Bra023810	A01	19285539	19288393	8	AT3G22760/*AtCPP*4
*BrCPP4b*	Bra033920	A05	16207472	16209714	6	AT3G22760/*AtCPP*4
*BrCPP5a*	Bra033919	A05	16191831	16195460	10	At3G22780/*AtCPP*5
*BrCPP5b*	Bra001884	A03	19146128	19152768	16	At3G22780/*AtCPP*5
*BrCPP6*	Bra036900	A01	12049577	12052924	8	AT4G14770/*AtCPP*6
*BrCPP7a*	Bra010348	A08	13239578	13242200	7	AT4G29000/*AtCPP*7
*BrCPP7b*	Bra011075	A01	4078631	4081317	6	AT4G29000/*AtCPP*7
*BrCPP7c*	Bra024171	A03	27178351	27180638	6	AT4G29000/*AtCPP*7
*BrCPP7d*	Bra025590	A04	8011846	8012463	1	AT4G29000/*AtCPP*7
*BrCPP8a*	Bra009859	A06	18346154	18348620	7	AT5G25790/*AtCPP*8
*BrCPP8b*	Bra036551	A09	2851635	2853619	4	AT5G25790/*AtCPP*8
*BrCPP8c*	Bra020513	A02	24310423	24316725	18	AT5G25790/*AtCPP*8
*BoCPP1*	Bol020837	C02	22859403	22861436	6	AT2G20110/*AtCPP*1
*BoCPP2*	Bol034129	Scaffold000040	1607390	1608218	3	AT3G04850/*AtCPP*2
*BoCPP3*	Bol034761	C01	32915941	32917598	5	AT3G16160/*AtCPP*3
*BoCPP4*	Bol023280	C01	27868376	27869528	3	AT3G22760/*AtCPP*4
*BoCPP6*	Bol008191	C01	38274359	38274904	2	AT4G14770/*AtCPP*6
*BoCPP7a*	Bol019616	C01	5441257	5443907	7	AT4G29000/*AtCPP*7
*BoCPP7b*	Bol021116	C08	16093123	16095690	6	AT4G29000/*AtCPP*7
*BoCPP8a*	Bol022330	C07	35441538	35444006	7	AT5G25790/*AtCPP*8
*BoCPP8b*	Bol032486	C09	3715173	3717272	5	AT5G25790/*AtCPP*8
*BoCPP8c*	Bol016381	C02	42997976	43000106	5	AT5G25790/*AtCPP*8
*BnA.CPP1b*	BnaA07g00040D	chrA07	21055	23400	6	AT2G20110/*AtCPP*1
*BnA.CPP2a*	BnaA03g28830D	chrA03	14033704	14036289	7	AT3G04850/*AtCPP*2
*BnA.CPP3b*	BnaA01g28180D	chrA01	19681341	19684544	6	AT3G16160/*AtCPP*3
*BnA.CPP4c*	BnaA05g16940D	chrA05	11718332	11720970	8	AT3G22760/*AtCPP*4
*BnA.CPP5b*	BnaA05g16930D	chrA05	11709193	11712979	10	At3G22780/*AtCPP*5
*BnA.CPP5d*	BnaA03g36790D	chrA03	18065426	18075292	16	At3G22780/*AtCPP*5
*BnA.CPP5e*	BnaA01g24520D	chrA01	16916341	16917772	5	At3G22780/*AtCPP*5
*BnA.CPP6a*	BnaA01g18960D	chrA01	10416162	10419574	8	AT4G14770/*AtCPP*6
*BnA.CPP6e*	BnaA03g15030D	chrA03	6929076	6937274	31	AT4G14770/*AtCPP*6
*BnA.CPP7a*	BnaA01g07920D	chrA01	3741875	3745039	7	AT4G29000/*AtCPP*7
*BnA.CPP7d*	BnaA08g30730D	chrA08_random	1678620	1681241	7	AT4G29000/*AtCPP*7
*BnA.CPP7f*	BnaAnng24750D	chrAnn_random	28633500	28634835	3	AT4G29000/*AtCPP*7
*BnA.CPP7g*	BnaAnng37830D	chrAnn_random	42872263	42873422	3	AT4G29000/*AtCPP*7
*BnA.CPP7h*	BnaA04g10170D	chrA04	8987674	8988291	1	AT4G29000/*AtCPP*7
*BnA.CPP8b*	BnaA06g27870D	chrA06	19167716	19170265	7	AT5G25790/*AtCPP*8
*BnA.CPP8d*	BnaA09g04480D	chrA09	2203990	2205985	4	AT5G25790/*AtCPP*8
*BnC.CPP1a*	BnaC07g00810D	chrC07	1116628	1119040	6	AT2G20110/*AtCPP*1
*BnC.CPP2b*	BnaC03g33970D	chrC03	20676845	20679238	6	AT3G04850/*AtCPP*2
*BnC.CPP3a*	BnaC01g35470D	chrC01	34864388	34866026	5	AT3G16160/*AtCPP*3
*BnC.CPP4a*	BnaCnng56700D	chrCnn_random	56576169	56579283	8	AT3G22760/*AtCPP*4
*BnC.CPP4b*	BnaCnng43510D	chrCnn_random	42545612	42548303	8	AT3G22760/*AtCPP*4
*BnC.CPP4d*	BnaCnng27640D	chrCnn_random	26215960	26218588	8	AT3G22760/*AtCPP*4
*BnC.CPP5a*	BnaCnng27630D	chrCnn_random	26211360	26214466	9	At3G22780/*AtCPP*5
*BnC.CPP5c*	BnaC03g42960D	chrC03	27663590	27671331	17	At3G22780/*AtCPP*5
*BnC.CPP5f*	BnaCnng43520D	chrCnn_random	42564973	42566124	4	At3G22780/*AtCPP*5
*BnC.CPP6b*	BnaC01g22810D	chrC01	16334181	16337785	8	AT4G14770/*AtCPP*6
*BnC.CPP6c*	BnaC03g18250D	chrC03	9349757	9353091	10	AT4G14770/*AtCPP*6
*BnC.CPP6d*	BnaC03g18050D	chrC03	9243783	9253263	31	AT4G14770/*AtCPP*6
*BnC.CPP7b*	BnaC01g09520D	chrC01	5562615	5570008	14	AT4G29000/*AtCPP*7
*BnC.CPP7c*	BnaC08g13260D	chrC08	18279478	18282187	7	AT4G29000/*AtCPP*7
*BnC.CPP7e*	BnaC07g41650D	chrC07	41430430	41432917	8	AT4G29000/*AtCPP*7
*BnC.CPP8a*	BnaC07g29080D	chrC07	33999748	34002214	7	AT5G25790/*AtCPP*8
*BnC.CPP8c*	BnaCnng48380D	chrCnn_random	47677091	47679547	5	AT5G25790/*AtCPP*8
*BnC.CPP8e*	BnaC02g40480D	chrC02	43513384	43515513	6	AT5G25790/*AtCPP*8

As we all know, *B. napus* is derived from natural hybridization and chromosome doubling of its two diploid progenitors, *B. rapa* and *B. oleracea*. Therefore, theoretically, the number of *CPP* genes in *B. napus* should be equal to the sum of the number of *CPP* genes in *B. rapa* and *B. oleracea*. In this study, 15 and 10 *CPP* genes were identified in *B. rapa* and *B. oleracea*, respectively, and the sum of *CPP* genes in the two species was much smaller than the number of *CPP* genes in *B. napus* (34). Specifically, the number of each *BnCPP* genes was greater than or equal to the sum of the corresponding homoeologous genes in the two diploid ancestral species (with one exception, *BnCPP8*; Supplementary Table 2). Compared with diploid species, the number of *CPP1-3* genes in *B. napus* did not change and the number of *CPP4-6* genes in both subgenomes of *B. napus* increased during allopolyploidization process. Statistically, the number of lost genes (2) was less than the number of increased genes (11) in *B. napus* (Supplementary Table 2). Therefore, the CPP gene family in *B. napus* expanded during the allopolyploidization process, although a small number of gene loss events occurred.

### The Duplication Mode and Synteny Analysis of *CPP* Genes

To explore the reasons for the expansion of CPP gene family in *B. napus*, we analyzed the duplication modes of *CPP* genes (Table 2 and Supplementary Figure 2). Gene duplication modes include whole genome duplication (WGD) and various single gene duplication, and the latter consists of four models, including tandem duplication (TD), proximal duplication (PD), transposed duplication (TRD) and dispersed duplication (DSD; Qiao et al., 2019). We found that *CPP* genes in *B. rapa*, *B. oleracea* and *B. napus* showed a large number of genome wide duplication, accounting for 60, 50, and 38.2%, respectively, which might be related to the WGD and WGT events of *Brassica* progenitor. For the four single gene duplication modes, the mechanism of the DSD is unclear (Qiao et al., 2019), so it was excluded from the following comparative analysis. We found that TRD was the most frequent mode (about 50%) in all three species, suggesting that this mode was important for the expansion of the CPP gene family. In addition, although WGD and TRD were the most frequent duplication modes of *CPP* genes in the three species, the frequency of TRD was higher than that of WGD only in the *CPP* genes of *B. napus*. Therefore, we speculated that TRD might greatly promote the expansion of CPP gene family in *B. napus* during allopolyploidization. Moreover, PD was observed in only one *CPP* gene in *B. rapa*, indicating that this duplication mode is not the main source of CPP gene family expansion in *B. rapa*, *B. oleracea* and *B. napus*. In addition, a total of 29 segmental duplicated gene pairs were detected in three *Brassica* species, and their *K*a/*K*s values were calculated to explore the selection pressure on *CPP* genes during evolution (Supplementary Table 3). The results showed that all the values were less than 1, indicating that this family was subjected to purification selection during evolution. The mean value of all *K*a/*K*s values was 0.42, which might indicate that the purification selection on *CPP* genes was not strong. One study suggested that *CPP* genes in plants might be subjected to purification selection only in the conserved domain (Yang et al., 2008). We also visualized these segmental duplication pairs and five duplication modes in Supplementary Figure 3.

**TABLE 2:** Five modes of *CPP* gene duplication in *B. napus* and its diploid progenitors.

Gene name	Gene ID	WGD	Single gene duplication			
			TRD	TD	PD	DSD
*BrCPP1*	Bra041042		√			√
BrCPP2	Bra001128		√			√
BrCPP3	Bra021165					√
BrCPP4a	Bra023810				√	√
*BrCPP4b*	Bra033920			√		√
*BrCPP5a*	Bra033919	√	√	√		
BrCPP5b	Bra001884	√	√			√
BrCPP6	Bra036900	√				√
BrCPP7a	Bra010348	√	√			√
BrCPP7b	Bra011075	√	√			√
*BrCPP7c*	Bra024171	√				√
*BrCPP7d*	Bra025590		√			√
*BrCPP8a*	Bra009859	√				√
BrCPP8b	Bra036551	√				√
BrCPP8c	Bra020513	√	√			√
BoCPP1	Bol020837		√			√
*BoCPP2*	Bol034129					
*BoCPP3*	Bol034761					√
*BoCPP4*	Bol023280	√	√			
*BoCPP6*	Bol008191		√			√
*BoCPP7a*	Bol019616	√	√			√
*BoCPP7b*	Bol021116	√				√
*BoCPP8a*	Bol022330	√				√
*BoCPP8b*	Bol032486	√				√
*BoCPP8c*	Bol016381	√	√			√
*BnC.CPP1a*	BnaC07g00810D		√			√
*BnA.CPP1b*	BnaA07g00040D		√			√
*BnA.CPP2a*	BnaA03g28830D					√
*BnC.CPP2b*	BnaC03g33970D					√
*BnC.CPP3a*	BnaC01g35470D		√			√
*BnA.CPP3b*	BnaA01g28180D					√
*BnC.CPP4a*	BnaCnng56700D		√			√
BnC.CPP4b	BnaCnng43510D			√		√
BnA.CPP4c	BnaA05g16940D			√		√
BnC.CPP4d	BnaCnng27640D		√			√
*BnC.CPP5a*	BnaCnng27630D		√			√
*BnA.CPP5b*	BnaA05g16930D	√		√		
*BnC.CPP5c*	BnaC03g42960D		√			√
BnA.CPP5d	BnaA03g36790D	√				√
BnA.CPP5e	BnaA01g24520D	√				√
BnC.CPP5f	BnaCnng43520D			√		√
BnA.CPP6a	BnaA01g18960D	√				√
BnC.CPP6b	BnaC01g22810D					√
BnC.CPP6c	BnaC03g18250D		√			√
BnC.CPP6d	BnaC03g18050D	√	√			√
BnA.CPP6e	BnaA03g15030D	√				√
*BnA.CPP7a*	BnaA01g07920D		√			√
BnC.CPP7b	BnaC01g09520D	√	√			√
BnC.CPP7c	BnaC08g13260D	√				√
BnA.CPP7d	BnaA08g30730D		√			√
BnC.CPP7e	BnaC07g41650D	√				√
BnA.CPP7f	BnaAnng24750D		√			√
BnA.CPP7g	BnaAnng37830D		√			√
BnA.CPP7h	BnaA04g10170D		√			√
BnC.CPP8a	BnaC07g29080D	√	√			√
BnA.CPP8b	BnaA06g27870D	√				√
BnC.CPP8c	BnaCnng48380D		√			√
BnA.CPP8d	BnaA09g04480D	√				√
BnC.CPP8e	BnaC02g40480D	√	√			√

**FIGURE 1:**
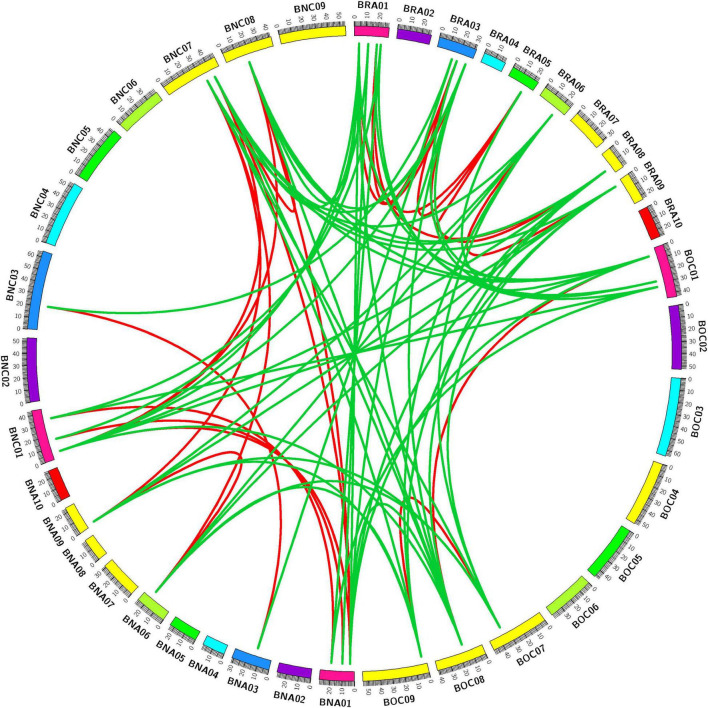
Synteny analysis of *CPP* genes in genomes of *B. rapa*, *B. oleracea*, and *B. napus*. Red lines and green lines separately linked the syntenic paralogs and orthologs. BRA01–10, ten chromosomes in *B. rapa*; BOC01–09, nine chromosomes in *B. oleracea*; BNA01–10, ten chromosomes in A_n_ sub-genomes in *B. napus*; BNC01–09, nine chromosomes in C_n_ sub-genomes in *B. napus*.

To better investigate the evolution of *CPP* genes among three *Brassica* species, we visualized the syntenic relationship of identified *CPP* genes. We found that *CPP* genes in these three *Brassica* species have a good syntenic relationship, and they were closely related in evolution (Figure 1). There were 8 *AtCPP* genes, 13 *BrCPP* genes, 7 *BoCPP* genes and 13 *BnCPP* genes, respectively, and these syntenic genes were mainly located in five conserved genome blocks F, H, Q, T, and U (Supplementary Table 4). In the three *Brassica* species, about 55.9% (33/59) of *CPP* genes retained some syntenic relationships between species. In addition, we found that the increase or loss of syntenic genes occurred in *B. napus* after allopolyploidization. For example, the number of *AtCPP2* syntenic genes in *B. rapa* and *B. oleracea* was 1 and 0 respectively, but the number of *AtCPP2* syntenic genes in *B. napus* increased to two. *AtCPP1* had one syntenic gene in *B. rapa* and *B. oleracea* respectively, but it was not found in *B. napus*. *AtCPP3* had one syntenic gene in both *B. rapa* and *B. oleracea*, which was all located in the LF subgenome (note: LF subgenome stands for less fractioned subgenome; Cheng et al., 2011, 2012), and these two genes were retained in *B. napus* and were also located in the two LF subgenomes. Therefore, we speculated that *AtCPP3* syntenic genes might play an irreplaceable role in the evolution and adaptation of these three *Brassica* species.

### Evolutionary Changes in Chromosomal Localization of *CPP* Genes

**FIGURE 2:**
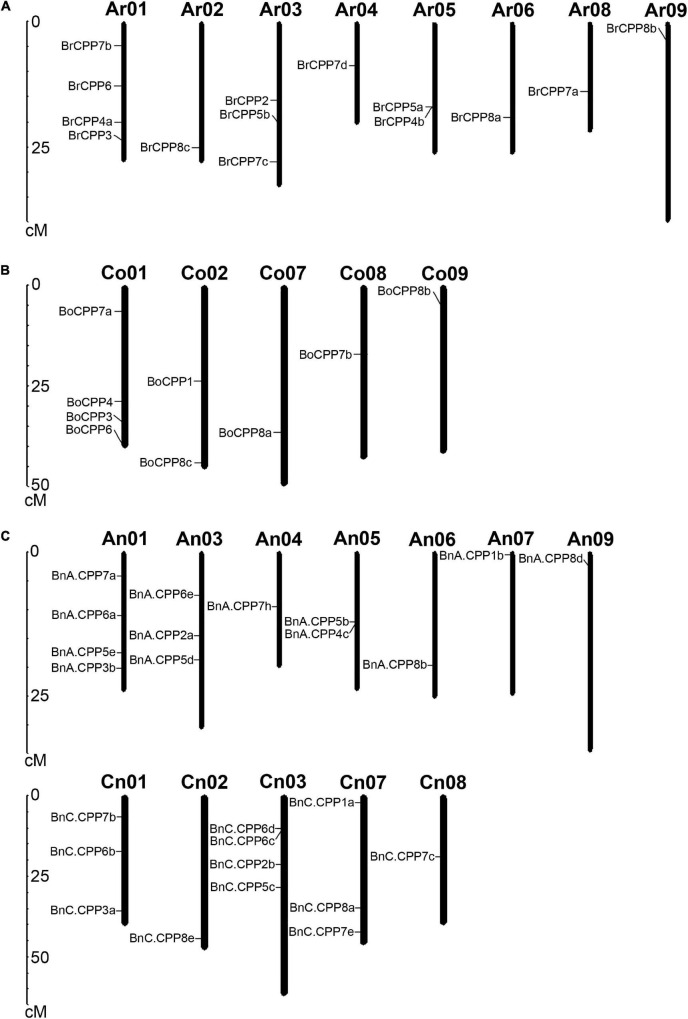
Chromosome distribution of CPP genes in *B. rapa* **(A)** and *B. oleracea* **(B)** and *B. napus* **(C)**. All genes located in unassembled scaffolds were not shown in this figure.

In addition to focusing on evolutionary changes in the number of *CPP* genes in *B. napus*, we also focused on the changes in the relative position of *CPP* genes on chromosomes. The number of *CPP* genes in A_n_ subgenome (16) was similar to that in the A_r_ genome (15), but the number of *CPP* genes in the C_n_ subgenome (18) was more than that in the C_o_ genome (10). Moreover, we noticed that most of *CPP* genes in the C_n_ subgenome (12 out of 18) experienced the TRD duplication event (Table 2), indicating that the number of *CPP* genes in C_n_ subgenome might expend after allopolyploidization, which might be related to the fact that more transposable elements were distributed in the C_n_ subgenome (Chalhoub et al., 2014). In addition, another reason for this phenomenon might be the species-specific loss of *CPP* genes in C_o_ genome during evolution. We further analyzed the chromosomal location of *CPP* genes, and a total of 25 *BnCPP* genes, 14 *BrCPP* genes and 9 *BoCPP* genes have been accurately mapped to the specific location of the chromosome (Figure 2). The remaining *CPP* genes have not been located in the chromosome due to the relatively low degree assembly of the reference genome. *CPP* genes were not found on 7 chromosomes (A_n_02, A_n_08, A_n_10, C_n_04-06, C_n_09), 2 chromosomes (A_r_07, A_r_10) and 4 chromosomes (C_o_03-06) in *B. napus*, *B. rapa* and *B. oleracea*, respectively. By comparing the distribution of *CPP* genes on A_n_ and A_r_ subgenome/genome, we found a similar pattern, that is, 4, 3, and 2 *CPP* genes were distributed on chromosome 1, 3 and 5, respectively, while the remaining *CPP* genes were distributed on other chromosomes as a single form. However, the distribution of *CPP* genes on C_n_ and C_o_ subgenome/genome was significantly different. In addition, about 71.4% (10/14) of *CPP* genes in A_r_ genome of *B. rapa* did not change their relative positions in chromosomes after allopolyploidization, while up to 44.4% (4/9) of *CPP* genes in C_o_ genome of *B. oleracea* maintained their original relative positions in chromosomes after allopolyploidization. This phenomenon was consistent with previous studies about the other gene families in *B. napus* (Li et al., 2019a,b), which implied that there might be more chromosomal rearrangement or homologous exchange events occurring in regions associated with these gene family members in the C_n_ subgenome than in the A_n_ subgenome.

### Evolutionary Changes in Gene Structure of *CPP* Genes

**FIGURE 3:**
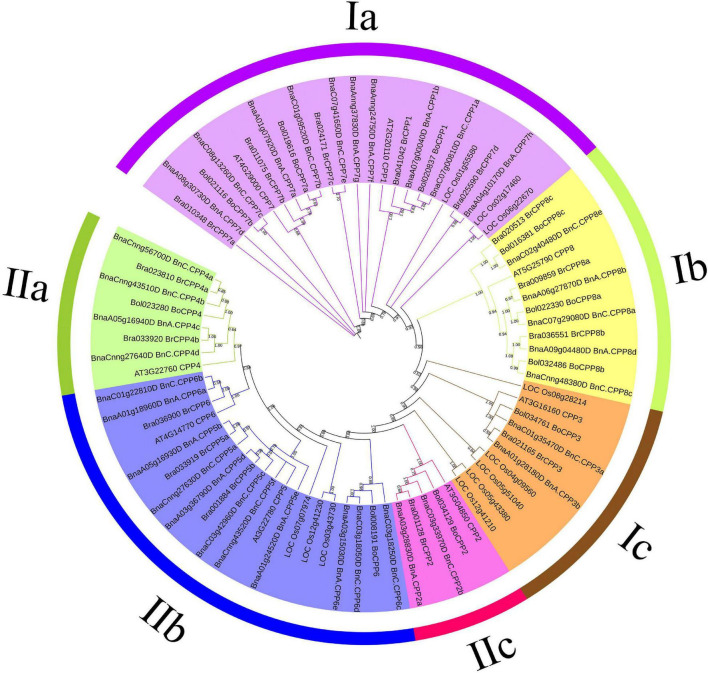
Phylogenetic tree of CPP proteins in *B. napus, B. rapa, B. oleracea, Arabidopsis thaliana*, and *Oryza sativa*. The prefixes Bra, Bol, Bna, AT, and LOC stand for *B. rapa*, *B. oleracea*, *B. napus*, *A. thaliana* and *O. sativa*, respectively. Only bootstrap values greater than 50% were displayed. All CPP proteins were divided into two clades (I,II) and six subclades (Ia-c,IIa-c).

We wanted to know what evolutionary changes had taken place in the gene structure of *CPP* genes after allopolyploidization. To better analyze these changes in terms of the evolutionary relationship, we first conducted phylogenetic analysis of the CPP gene family. CPP proteins of typical monocotyledon rice and dicotyledon Arabidopsis were used as reference proteins, and a phylogenetic tree was constructed based on 78 CPP protein sequences, among which there were 8, 11, 15, 10, and 34 CPP proteins in Arabidopsis, rice, *B. rapa*, *B. oleracea* and *B. napus*, respectively (Figure 3). These CPP proteins can be clearly divided into two clades (I, II) and six subclades (Ia-c, IIa-c), which was consistent with CPP phylogenetic tree in other species (Zhou et al., 2018). We found that the CPP protein of Arabidopsis was present in all six subclades, whereas the CPP protein of rice was found in only three subclades (Ia, Ic, IIb), which might indicate that the evolution of CPP family has gradually diverged after the differentiation of monocotyledon and dicotyledon.

**FIGURE 4:**
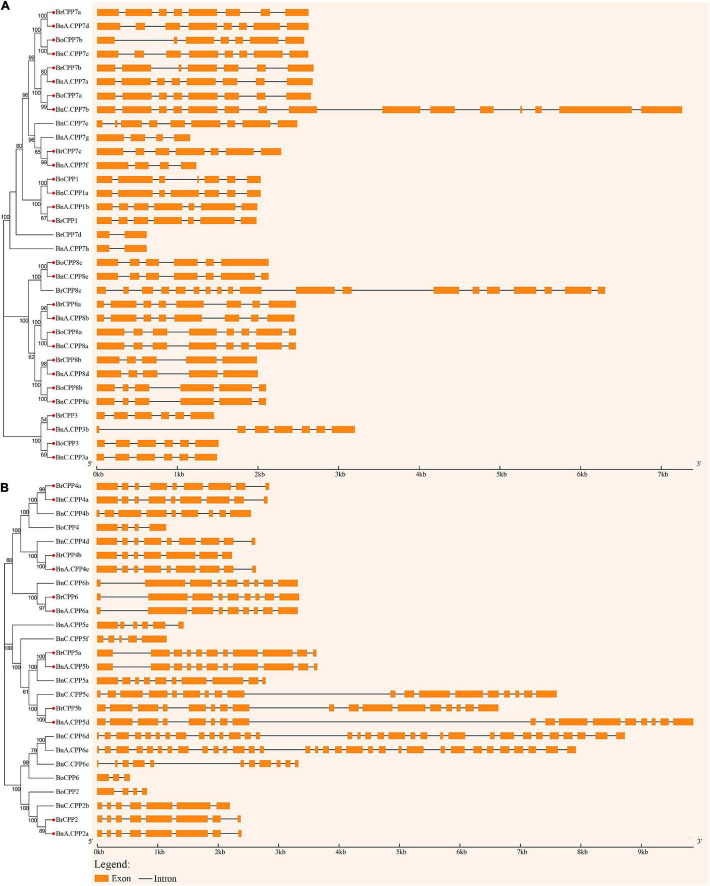
Intron-exon structure of the *CPP* genes in *B. napus* and diploid progenitors. The genes on two clades, I **(A)** and II **(B)**, of the phylogenetic tree were shown separately. The red circle indicates orthologs with potential direct evolutionary relationship.

Next, we analyzed the structure of *CPP* genes in *B. napus* and its two diploid progenitors. Compared with many other gene families, members of the CPP gene family have a high diversity of gene structure (Figure 4). We compared the structural diversity of genes in two clades of the phylogenetic tree, and found that both the structural diversity and the mean number of introns of *CPP* gene in clade II were higher than those in clade I, indicating that the structure of *CPP* genes in clade I may be more conserved than that in clade II. In addition, we found that *BnCPP* genes had the highest intron number and structural diversity, while *BoCPP* genes had the lowest intron number and structural diversity, indicating that *CPP* genes in *B. napus* evolved toward increasing intron number and structural diversity after allopolyploidization. To further explore the evolutionary changes in the structure of *CPP* genes after allopolyploidization, we identified 20 homoeologous gene pairs with potential direct evolutionary relationship and conducted comparative analysis of their gene structure according to Li et al. (2019a; 2019b). Among the 20 homoeologous gene pairs, the intron number of 13 gene pairs (65%) was the same between *B. napus* and its diploid progenitors, which suggested that *CPP* genes are conserved to a certain extent at the DNA level after allopolyploidization. Moreover, among the remaining 7 gene pairs, there were 6 gene pairs showed the number of introns in *BnCPP* gene was greater than that in *BrCPP* or *BoCPP* genes. These results indicated that intron increase or loss occurred in some *CPP* genes after allopolyploidization, and the phenomenon of intron increasing was more common.

### The CXC Domain and Motif Analysis of CPP Proteins

Analysis of CXC domains in *B. napus* and its progenitors showed that most CPP proteins in the three *Brassica* species contained one or two conserved CXC domains, just like CPP proteins in other species (Supplementary Figure 4). However, there were four CPP proteins (BnC.CPP5c, BrCPP5b, BnA.CPP5d, BnC.CPP6c) had four CXC domains, which have not been observed in CPP proteins of other species. This phenomenon might be related to the occurrence of homoeologous exchange events during allopolyploidization (Chalhoub et al., 2014). The alignment results of CXC domains in all identified CPP proteins showed that the two CXC domains and the short-conserved sequence RNPXAFXPK between them were very conserved, and only a few CPP proteins had amino acid deletion or replacement at these locations (Supplementary Figure 5). The motif analysis showed that 6 motifs existed in almost all CPP proteins, which were motifs 1–5 and 9 (Supplementary Figure 6). By analyzing the sequences of these motifs, we found that motifs 2, 4, and 9 were associated with the N-terminal CXC domain of CPP proteins, motif 5 was associated with a short-conserved sequence between two CXC domains, and motifs 1 and 3 were associated with the C-terminal CXC domain of CPP proteins. The motifs of proteins encoded by 20 homoeologous gene pairs with potential direct evolutionary relationship were compared, and we found 15 (75%) BnCPP proteins maintained the similar motif type and arrangement pattern of BrCPP/BoCPP proteins, indicating that the motif of CPP proteins were relatively conservative after allopolyploidization.

### Physicochemical Properties and Subcellular Localization Prediction of CPP Protein

We predicted the physicochemical properties of CPP proteins in three *Brassica* species (Table 3), and the results were as follows. The average lengths of BnCPP, BrCPP and BoCPP proteins were 603, 592, and 381 aa, respectively, and the average molecular weights were 66.2, 65.0, and 41.5 kDa, respectively, which means that the average length and average molecular weight of BnCPP proteins were higher than those of its two progenitors. Furthermore, BnC.CPP7b and BoCPP7a were homoeologous gene pairs with potential direct evolutionary relationship, and we found that the protein sequence length of BnC.CPP7b (1408 aa) is much longer than that of BoCPP7a (578 aa), indicating that the encoding region of *CPP* gene might have increased after allopolyploidization. It was noteworthy that the length of 6 CPP proteins over 1000 aa were found in three *Brassica* species, and 5 of them were belonging to clade II in phylogenetic tree (Figure 3). In the three *Brassica* species, the instability index of all CPP proteins was higher than 40, and the GRAVY of all CPP proteins (except BoCPP2) was lower than 0, indicating that almost all CPP proteins were unstable hydrophilic proteins. The results showed that 44.12% of BnCPP, 53.33% of BrCPP and 30% of BoCPP proteins were acidic proteins (pI < 7), indicating that the ratio of acidic CPP protein in *B. napus* was between the two diploids. In addition, except for subclade Ic, acidic CPP proteins were found in all the other clades, suggesting that this clade might have diverged from other clades in the process of evolution. Subsequently, we predicted the subcellular localization of CPP proteins in the three *Brassica* species (Table 3). The results showed that the subcellular localization of CPP protein in three *Brassica* species was the same as that in other species (Song et al., 2016; Zhou et al., 2018). Most CPP proteins in *B. napus* and its diploid progenitors were predicted to be located in the nucleus, which may be consistent with the function of CPP proteins as transcription factors.

**TABLE 3:** The physicochemical parameters and subcellular localization prediction of CPP proteins in *B. napus* and its diploid progenitors.

Protein	Protein	MW	pI	Instability	Gravy	Subcellular
name	length (aa)	(kDa)		index		localization
BnA.CPP1b	506	54.25883	7.13	52.07	–0.618	Nucleus
BnA.CPP2a	522	57.31421	4.95	60.01	–0.435	Nucleus
BnA.CPP3b	340	38.12622	7.93	53.21	–0.607	Nucleus
BnA.CPP4c	574	62.93197	8.31	67	–0.624	Nucleus
BnA.CPP5b	748	82.16356	8.04	60.97	–0.629	Nucleus
BnA.CPP5d	1216	133.79573	7.8	65.78	–0.657	Nucleus
BnA.CPP5e	286	31.9237	8.62	58.3	–0.329	Extracell
BnA.CPP6a	647	71.22856	5.5	68.84	–0.719	Nucleus
BnA.CPP6e	1300	142.52083	6.86	46.9	–0.595	Nucleus
BnA.CPP7a	583	62.11057	8.63	53.37	–0.635	Nucleus
BnA.CPP7d	573	61.27621	7.72	58.92	–0.667	Nucleus
BnA.CPP7f	287	31.24956	9.17	53.6	–0.614	Nucleus
BnA.CPP7g	265	28.59231	9.1	53.52	–0.689	Nucleus
BnA.CPP7h	142	15.87176	6.06	45.94	–0.585	Cytoplasm
BnA.CPP8b	510	56.64173	6.02	54.58	–0.922	Nucleus
BnA.CPP8d	455	50.42734	6.55	53.2	–0.853	Nucleus
BnC.CPP1a	495	53.42396	7.44	50.38	–0.623	Nucleus
BnC.CPP2b	543	60.0745	5.07	59.62	–0.357	Chloroplast
BnC.CPP3a	328	36.63148	7.68	53.09	–0.626	Nucleus
BnC.CPP4a	604	66.34732	6.9	67.44	–0.683	Nucleus
BnC.CPP4b	596	65.46938	7.44	66.98	–0.626	Nucleus
BnC.CPP4d	549	60.17399	8.33	67.36	–0.576	Nucleus
BnC.CPP5a	679	74.87134	6.67	58.09	–0.654	Nucleus
BnC.CPP5c	1241	137.16818	6.84	62.82	–0.69	Chloroplast
BnC.CPP5f	264	29.9765	9.58	58.62	–0.671	Nucleus
BnC.CPP6b	657	71.95252	5.49	65.58	–0.656	Nucleus
BnC.CPP6c	323	35.73944	8.67	58.07	–0.827	Nucleus
BnC.CPP6d	1296	141.3965	6.87	46.71	–0.585	Nucleus
BnC.CPP7b	1408	155.07617	6.42	58.2	–0.744	Vacuole
BnC.CPP7c	566	60.36419	7.44	56.95	–0.639	Nucleus
BnC.CPP7e	558	60.71411	8.35	64.69	–0.745	Nucleus
BnC.CPP8a	519	57.41975	6.54	51.48	–0.888	Nucleus
BnC.CPP8c	458	50.79463	7.09	51.89	–0.898	Nucleus
BnC.CPP8e	478	52.64484	6.22	58.45	–1.014	Nucleus
BoCPP1	385	41.55645	5.7	51.38	–0.677	Nucleus
BoCPP2	175	19.54062	7.36	40.42	0.066	Cytoplasm
BoCPP3	330	36.89163	8.1	52.75	–0.693	Nucleus
BoCPP4	265	29.24992	7.94	69.21	–0.631	Nucleus
BoCPP6	130	14.11321	8.94	50.85	–0.606	Nucleus
BoCPP7a	578	61.33569	8.56	55.56	–0.635	Nucleus
BoCPP7b	464	49.00643	7.17	59.94	–0.597	Nucleus
BoCPP8a	518	57.36174	6.66	50.39	–0.895	Nucleus
BoCPP8b	457	50.72355	7.09	51.8	–0.904	Nucleus
BoCPP8c	504	55.66243	6.27	57.74	–0.95	Nucleus
BrCPP1	503	53.9774	7.14	52.58	–0.639	Nucleus
BrCPP2	521	57.32656	5.06	62.28	–0.369	Nucleus
BrCPP3	326	36.54725	7.7	51.68	–0.708	Nucleus
BrCPP4a	592	64.90159	6.17	66.52	–0.651	Nucleus
BrCPP4b	575	63.43283	8.66	70.18	–0.539	Chloroplast
BrCPP5a	748	82.23362	7.88	61.8	–0.628	Nucleus
BrCPP5b	1223	134.46711	6.74	63.78	–0.662	Nucleus
BrCPP6	645	71.09954	5.51	68.35	–0.693	Nucleus
BrCPP7a	574	61.44543	7.93	59.66	–0.664	Nucleus
BrCPP7b	528	56.58224	8.51	54.86	–0.729	Nucleus
BrCPP7c	552	60.40711	8.84	52.84	–0.77	Nucleus
BrCPP7d	142	15.87176	6.06	45.94	–0.585	Cytoplasm
BrCPP8a	509	56.50368	5.81	53.9	–0.892	Nucleus
BrCPP8b	456	50.6566	6.68	52.3	–0.861	Nucleus
BrCPP8c	990	110.14645	5.73	50.22	–0.592	Nucleus

### Prediction of *Cis*-Acting Elements in the Promoter Region of *CPP* Genes

**FIGURE 5:**
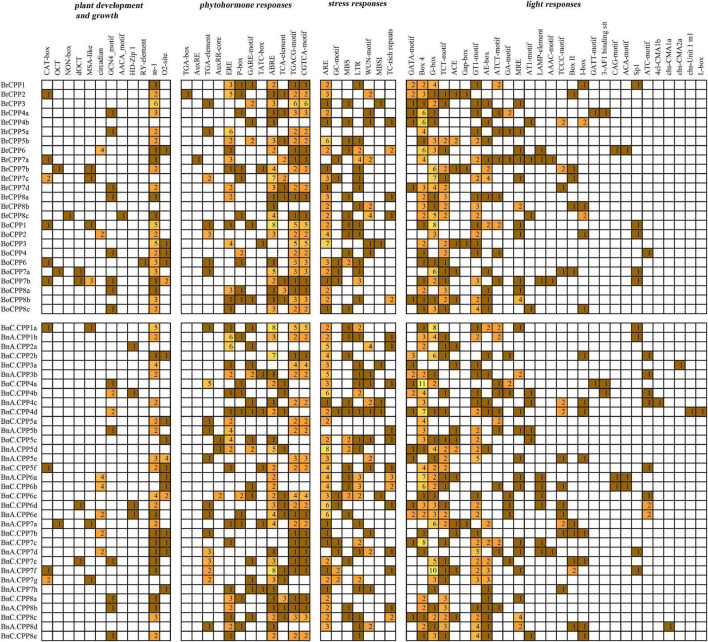
*Cis*-acting elements on promoters of *CPP* genes from *B. napus* and diploid progenitors.

We predicted the *cis*-acting elements in the upstream promoter region of *CPP* genes in three *Brassica* species (Figure 5). Fifty-nine *cis*-acting elements related to plant development and growth, phytohormone responses, stress responses and light responses were identified in the promoter region of *CPP* genes in *B. napus* and its progenitors. Among them, the largest number of *cis*-elements were associated with light responses. Seven identical stress response elements were identified in each of the three *Brassica* species, and the antioxidant response element (ARE) was found in the promoter region of 52 *CPP* genes, which was the most common type of *cis*-acting elements. We hypothesized that these stress-related elements, especially the antioxidant elements, existed in two diploid *Brassica* progenitors and were entirely preserved during allopolyploidization. We also found some elements related to development and growth that could regulate cell cycle (MSA-like) and affect expression of meristem (CAT-box), and meanwhile, *CPP* genes have been shown to participate in the coordination of cell cycle and fate (Wang et al., 2018). In addition, a large number of hormone-responsive elements have been found, including methyl jasmonate response elements (CGTCA-motif), abscisic acid response elements (ABRE) and salicylic acid response elements (TCA-element). We further analyzed and compared the *cis*-acting elements of the promoter regions of 20 homoeologous gene pairs mentioned above, and the results showed that there were four gene pairs (*BrCPP7b* and *BnA.CPP7a*, *BoCPP1* and *BnC.CPP1a*, *BrCPP8a* and *BnA.CPP8b*, *BoCPP8a* and *BnC.CPP8a*) have the same *cis*-acting element type, indicating that the *cis*-acting elements of the promoter regions in these *CPP* genes were conserved during allopolyploidization. Moreover, eight *BnCPP* genes were found to have more *cis*-element types or numbers than their diploid progenitors, which might contribute to the regulatory role of *CPP* gens in the genetics, development and evolution of *B. napus*.

### Analysis of the Expression Patterns of *CPP* Genes

**FIGURE 6:**
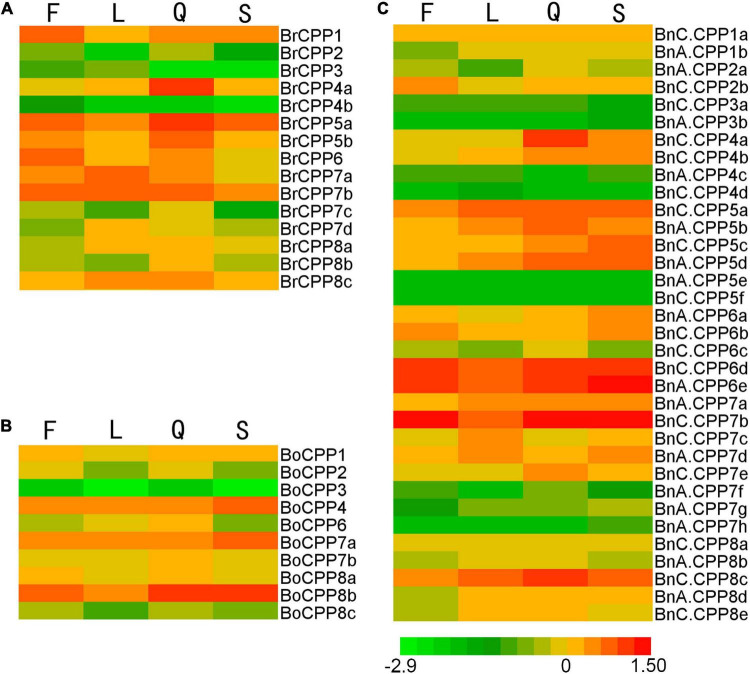
Expression patterns of CPP genes in flowers (F), leaves (L), siliques (Q) and stems (S) from *B. rapa* **(A)**, *B. oleracea* **(B)**, and *B. napus* **(C)**.

To explore the expression patterns of *CPP* genes, we analyzed the expression patterns of *CPP* genes in four organs (stems, leaves, flowers and siliques) in *B. napus* and its diploid progenitors (Figure 6). *CPP* genes were widely expressed in the four organs, suggesting that the CPP gene family might have multiple biological functions and might play roles in different organs. Two *BnCPP* genes (*BnA.CPP5e* and *BnC.CPP5f*) were not expressed in the four organs, suggesting that they might not play a role in these organs or might be expressed in specific time and space. The expression patterns of different *CPP* genes were various in the four organs. For example, the expression level of *BrCPP1* was relatively high in flowers, while the expression level of *BrCPP4a* and *BnC.CPP4a* was relatively high in siliques. Based on the expression data of 20 homoeologous gene pairs with potential direct evolutionary relationship, we compared the expression patterns between *BnCPP* and *BrCPP*/*BoCPP* genes to explore the evolutionary changes of *CPP* gene expression during allopolyploidization. We found most of these gene pairs showed different expression patterns in these organs. The expression of some *BnCPP* genes and their ancestral homoeologous genes in some organs was completely opposite. For example, the expression levels of *BrCPP1* and *BoCPP8a* were the highest in flowers, while the expression levels of *BnA.CPP1b* and *BnC.CPP8a* were the lowest in flowers. Similarly, the expression level of *BoCPP8c* was the lowest in leaves, while the expression level of *BnC.CPP8e* was the highest in leaves. In addition, some *CPP* genes were expressed at roughly the same level in different organs, while the expression of their homoeologous genes were significantly different in these organs. For example, the expression level of *BoCPP7a* in leaves and siliques was identical, while the expression level of *BnC.CPP7b* in leaves was significantly lower than that in siliques. Moreover, one gene of the homoeologous pairs was expressed and the other was not expressed in some organs. For example, *BnA.CPP3b* was expressed only in stems, while its homoeologous gene *BrCPP3* was expressed in flowers and leaves, but not in stems. These results indicated that the expression patterns of *CPP* genes might be changed in the process of allopolyploidization. By comparing the expression levels of 13 homoeologous gene pairs with the same gene structure in the four organs, it was found that the majority of them had different expression patterns, that is, the gene expression might change though there was no significant change at the DNA level, which might be related to the regulation of gene expression during allopolyploidization.

To study the expression bias of *CPP* genes in different organs of allotetraploid *B. napus*, 20 homoeologous gene pairs were classified into eight groups (*CPP1*–*CPP8*) based on their homology. There were four groups (*CPP2*, *CPP4*–*CPP6*) in which the ancestor genes were not present in both *B. rapa* and *B. oleracea*, so they could not be analyzed in this way. Finally, four groups of genes (*CPP1*, *CPP3*, *CPP7*, and *CPP8*) were used for the gene expression bias analysis. The results showed that the expression bias of *BnCPP* genes was inconsistent in different organs (Supplementary Table 5). Specifically, the expression of *BnCPP8* was biased toward *B. rapa* in all four organs. The expression of *BnCPP3* was biased toward *B. rapa* in flowers, leaves and siliques, while which was biased toward *B. oleracea* in stems. The expression of *BnCPP7* was also biased toward *B. rapa* in three organs (flowers, stems and siliques), and was biased toward *B. oleracea* in leaves. The expression of *BnCPP1* was biased toward *B. rapa* only in leaves, and was biased toward *B. oleracea* in the other three organs (flowers, stems and siliques). We found that 75% of *BnCPP* gene expression was biased toward *B. rapa* in flowers, leaves and siliques, while this proportion was only 50% in stems.

### *CPP* Gene Expression Changes of Three *Brassica* Species Under Salt Stress

**FIGURE 7:**
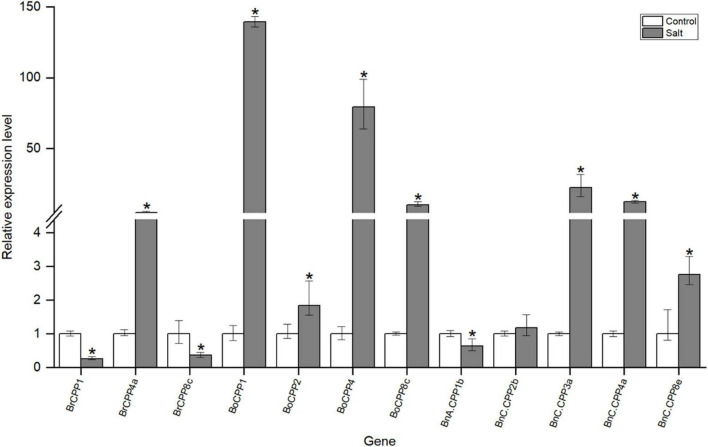
Expression of *CPP* genes in leaves of *B. rapa, B. oleracea*, and *B. napus*. Control: the control group; Salt: salt treatment group; “*” represents a significant difference between the two groups.

Previous studies have shown that CPP gene family can respond to a variety of abiotic stresses, such as low temperature and drought stress. To investigate whether the CPP gene family in three *Brassica* species responds to salt stress, we analyzed the expression of some *CPP* genes under salt stress by quantitative real-time PCR (qRT-PCR). *CPP* genes whose expression was not detected in the experimental material were not analyzed in further study, and we finally selected 12 *CPP* genes for the qRT-PCR experiment (Supplementary Table 1). The expression levels of 11 *CPP* genes were significantly changed after salt stress, suggesting that they might be involved in the response to salt stress (Figure 7). Further analysis showed that the expression levels of 8 *CPP* genes increased significantly after salt stress, while the expression levels of 3 *CPP* genes decreased significantly. These results suggest that the CPP gene family might adapt to salt stress through two different response mechanisms (positive and negative), and positive regulation might be the main regulatory mode. It was noted that the expression levels of some *CPP* genes increased sharply after salt stress. For example, the expression levels of *BoCPP1* and *BoCPP4* were upregulated by 140 and 80 times, respectively, and their expression levels were much higher than those of other up-regulated genes. Therefore, it was speculated that these two *BoCPP* genes might play dominant roles in salt stress response.

In addition, there were three homoeologous gene pairs that have potential direct evolutionary relationship (*BrCPP1* and *BnA.CPP1b*, *BrCPP4a* and *BnC.CPP4a*, *BoCPP8c* and *BnC.CPP8e*) among the 11 *CPP* genes whose expression levels changed significantly after salt stress, and the changes of their expression levels after salt stress were compared and analyzed. We found that the expression of the three *BnCPP* genes and their homoeologous genes in two diploid progenitors showed the same trend after salt stress. In particular, the expression of *BrCPP1* and *BnA.CPP1b* was down-regulated after salt stress, and the expressions of *BrCPP4a* and *BnC.CPP4a* and *BoCPP8c* and *BnC.CPP8e* were up-regulated after salt stress. However, the expression levels of these *BnCPP* genes and their corresponding homoeologous genes in two diploid progenitors were different in degree. For example, the expression of *BnC.CPP4a* was up-regulated about 12.5 times, while the expression of *BrCPP4a* was up-regulated only about 4.8 times after salt stress, and the expression of *BnC.CPP8e* increased 2.8 times, while the expression of *BoCPP8c* increased 10.3 times after salt stress. These results suggested that the response degree of *CPP* genes to salt stress might change after allopolyploidization.

### Physiological Changes of Three *Brassica* Species Under Salt Stress

**FIGURE 8:**
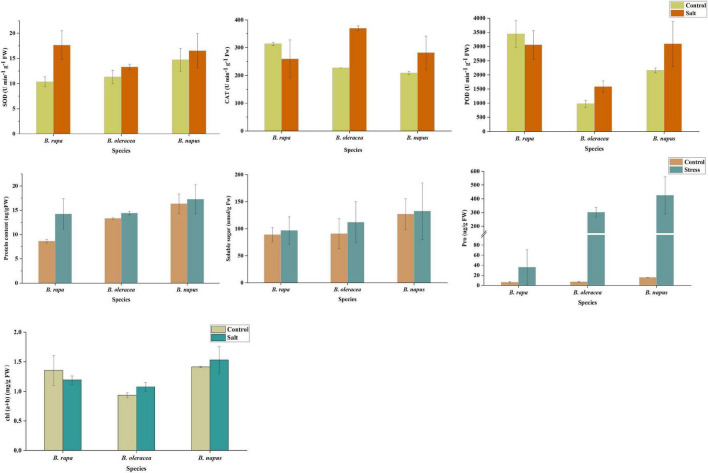
The physiological responses of *B. napus* and diploid progenitors to salt stress.

The superoxide dismutase (SOD) activity of the three *Brassica* species increased after salt stress, among which the SOD activity of *B. rapa* increased the most, which was 1.7 times of the control, and the SOD activity of *B. oleracea* and *B. napus* was 1.18 times and 1.12 times of the control, respectively (Figure 8). After salt stress, peroxidase (POD) activity of *B. oleracea* and *B. napus* increased by 1.62 and 1.43 times, respectively, while POD activity of *B. rapa* decreased. Similarly, after salt stress, POD activity of *B. oleracea* and *B. napus* increased by 1.63 and 1.35 times, respectively, while catalase (CAT) activity of *B. rapa* decreased. Therefore, the activities of all three enzymes were increased to remove reactive oxygen in *B. oleracea* and *B. napus* after salt stress, while only the activity of SOD was increased in *B. rapa*, and the activities of the other two enzymes were restricted. These results suggested that the response of antioxidant enzyme systems in three *Brassica* species to salt stress might be different.

Plants can also respond to adverse conditions by accumulating osmotic substances. Soluble protein accumulation occurred in all three *Brassica* species after salt stress, which was most obvious in *B. rapa* (Figure 8). For details, after salt stress, the soluble protein content of *B. rapa* was 1.65 times that of the control, while which of *B. oleracea* and *B. napus* was 1.08 and 1.05 times that of the control, respectively. The soluble sugar content of three *Brassica* species increased after salt stress, and which of *B. oleracea* increased most. The soluble sugar content of *B. oleracea* was 1.23 times that of the control, and which of *B. rapa* and *B. napus* was 1.09 and 1.04 times that of the control, respectively. In addition, we observed an interesting phenomenon that the content of proline in the three *Brassica* species increased sharply after salt stress, and the content of proline in *B. rapa*, *B. oleracea*, and *B. napus* were 4.7, 40.9 and 26.3 times higher than that of the control, respectively. These results indicated that these substances played the corresponding osmotic regulation functions to alleviate the salt stress injury of *B. napus* and its progenitors. Moreover, after salt stress, chlorophyll content in leaves of *B. rapa* decreased, but which of *B. oleracea* and *B. napus* increased, suggesting that the three species might have different salt-stress response mechanisms.

## Discussion

Polyploidization of plants can lead to the enhancement of their environmental adaptability (Segraves, 2017), and the formation and evolution of polyploids are often accompanied by series changes in plant morphology, physiology, genetics and epigenetics (Soltis et al., 2016). A large number of studies have been conducted on the large-scale genetic and epigenetic changes of plants after allopolyploidization, but there were few studies focused on the molecular basis of environmental adaptability on allopolyploids from the perspective of gene family. The allopolyploid *B. napus* and its two diploid progenitors (*B. rapa* and *B. oleracea*) was an appropriate system to study the scientific questions about allopolyploidization. As the transcription factor gene family, CPP gene family played a role in stress response of plants, and this gene family was not reported in *Brassica* species. In this study, the characteristics and the salt response of CPP gene family in *B. napus* and its progenitors were compared, which was conducive to exploring the genetic evolution characteristics of CPP gene family and detecting the potential advantageous molecular basis of stress response in allopolyploid *B. napus*.

### Expansion of *CPP* Gene Family in Allopolyploid *Brassica napus*

The phenomenon of gene family expansion is widespread in the process of plant evolution. For example, the majority of gene families (about 80%) in *Arabidopsis thaliana* showed expansion in their evolutionary course (Lespinet et al., 2002). In this study, 15, 10, and 34 *CPP* genes were identified in *B. rapa*, *B. oleracea*, and *B. napus*, and the number of genes in this three *Brassica* species can be analyzed as follows. *Brassica* and *Arabidopsis* diverged from a common ancestor about 14 million years ago (MYA; Song et al., 2020). All 8 *CPP* genes in Arabidopsis had corresponding homoeologous genes in *B. rapa*, *B. oleracea*, and *B. napus*, indicating that the progenitors of *Brassica* retained these *CPP* genes completely after being differentiated from the evolutionary lineage of *Arabidopsis*. After separating from the evolutionary lineage of *Arabidopsis*, the progenitors of *Brassica* underwent a unique whole genome triplication (WGT) event and divided into two diploid species *B. rapa* and *B. oleracea* about 3 MYA (Song et al., 2020). Therefore, in theory, if the progenitors of *Brassica* had not lost genes after the WGT event, there should be three times as many *CPP* genes in *B. rapa* and *B. oleracea* as in Arabidopsis. However, we found that many *CPP* genes in Arabidopsis have less than three homoeologous genes in diploid *B. rapa* and *B. oleracea* (Table 1). Therefore, due to the WGT events, the CPP gene family in *Brassica* expands compared to *Arabidopsis*, and at the same time, gene loss events occur in large numbers. In addition, the allotetraploid *B. napus* was formed by hybridization and genome doubling (i.e., allopolyploidization) of two diploid *B. rapa* and *B. oleracea* about 7,500 years ago (Chalhoub et al., 2014). Theoretically, the number of CPP gene family members in *B. napus* should be equal to the sum of the number of CPP members in the diploid *B. rapa* and *B. oleracea*. In this study, we found that the total number of CPP gene family members in *B. napus* was more than the sum of the two diploid progenitor species (34 > 15 + 10), that is, the CPP gene family also expanded in *B. napus* during allopolyploidization. Moreover, loss of *CPP* genes, such as *CPP8* homoeologues gene (5 < 3 + 3), was also found during allopolyploidization. From the above analysis, it can be concluded that the CPP gene family expanded in *B. napus* during evolution, despite the occurrence of gene loss events.

The main duplication modes that cause gene family expansion might be different. Analysis of gene duplication modes in gene families can provide explanations for the expansion of these gene families (Liang et al., 2017). Cannon et al. (2004) studied the duplication modes of 50 gene families in *Arabidopsis thaliana*, and found that the tandem duplication and segmental duplication were dominant in many gene families. In this study, the duplication modes of the *CPP* genes were studied, and we found that in addition to a large number of WGD, many TRD were detected in *CPP* genes of three *Brassica* species. In addition, unlike the two diploid *Brassica* species, the proportion of TRD in *CPP* genes was higher than that of WGD in *B. napus*. Therefore, it was speculated that TRD may be an important driving force for the expansion of CPP gene family in *B. napus* during allopolyploidization.

### Intron Acquisition Events Were Observed in *BnCPP* Genes After Allopolyploidization

As an important part of structural genes in eukaryotes, the evolution and function of introns are particularly important. In the study of gene families, analyzing the gene structural changes (changes of the number of exon/intron) may help us to understand the evolutionary direction of gene families. In this study, a comparative analysis of the gene structure of 20 homoeologous gene pairs with potential direct evolutionary relationship was conducted. Our results showed that except for 13 homoeologous gene pairs that were highly conserved in intron number after allopolyploidization, the remaining 7 gene pairs showed differences in intron number, and 6 of them showed more introns in *BnCPP* than in the corresponding ancestral species *BrCPP*/*BoCPP*, indicating that most of the *CPP* genes with structural changes acquired new introns after allopolyploidization. Similar phenomena have been observed in other gene families of *B. napus* (Li et al., 2019a). Earlier studies have suggested that introns might bring heavy burdens to the organism. For example, on the one hand, intron splicing required a high biological cost (Jiang and Goertzen, 2011). On the other hand, because introns were not subjected to the pressure of natural selection, a large number of mutations were often accumulated in introns, and accumulation of harmful mutations might adversely affect the adaptation of the organism (Lynch, 2006). However, recent studies have consistently suggested that the presence of introns might provide various benefits to the genome and organisms (Mukherjee et al., 2018), of which several have been discovered. First, introns could be used to improve protein diversity through alternative splicing and the fractionation of exons (Lamond, 1999). Second, introns might lead to the production of non-coding RNAs that were involved in some regulatory processes (Ying and Lin, 2005). Third, introns could also play a positive mediating role to enhance/promote the expression of some genes, as researchers have found that genes with higher expression levels have more and longer introns than genes with lower expression levels in rice and *Arabidopsis thaliana* (Ren et al., 2006). In addition, introns might also play a positive role in transcriptional coupling and mRNA output (Maniatis and Reed, 2002). Therefore, the increase of introns in *BnCPP* genes after allopolyploidization might be beneficial to the genome evolution of *B. napus*, but the specific effects still need further studies to prove.

### The Expression Characteristics of *BnCPP* Genes Might Indicate the Evolutionary Advantage of *B. napus*

A series of genetic and epigenetic changes, occurring rapidly when polyploids were established, which not only help stabilize the plant genome, but also regulate the expression of genes in the plant (Pikaard, 2001). To understand whether gene expression changes occurred during the formation and evolution of allotetraploid *B. napus*, the expression pattern of *CPP* genes in three *Brassica* species was investigated in this study. The results showed that, on the one hand, except for two *BnCPP* genes, *CPP* genes were widely expressed in four organs of the three *Brassica* species, suggesting that the *CPP* genes might be indispensable in the genetic, developmental and evolutionary process of three species. On the other hand, we also observed various changes in *CPP* gene expression during allopolyploidization. For example, most gene pairs, despite having the same gene structure, showed divergence in their expression patterns. Therefore, it was speculated that there might be neofunctionalization or subfunctionalization of *CPP* genes during allopolyploidization, and these changes might have beneficial effects on the evolution of CPP gene family in *B. napus*. Moreover, the differentiation of gene function might also reduce the risk of redundant genes being eliminated. In addition, we found that the *BnCPP* gene showed different bias expression, which was different in four organs. For example, the expression of *BnCPP3* and *BnCPP7* in three organs were biased toward *B. rapa*, while the expression of *BnCPP1* in three organs was biased toward *B. oleracea*. It was speculated that such a variety of biased gene expression might help allopolyploid *B. napus* adapt better to the environment (Samans et al., 2017).

Previous studies have suggested that *CPP* genes may play a role in response to various abiotic stresses. For example, most *CPP* genes in the roots of soybean can respond to heat stress, and many *CPP* genes in the leaves of cucumber show different response patterns and can respond to various stresses such as low temperature, drought, ABA and salt (Zhang et al., 2015; Zhou et al., 2018). *B. napus*, as an important crop, may be affected by various adverse environment during its growth, among which salting environment is one of the main adverse environments. In this study, we investigated the expression of some *CPP* genes in *B. napus* and its progenitors under salt stress. The results showed that *CPP* gene expression levels were significantly changed in *B. napus* and its two progenitors after salt stress, suggesting that *CPP* genes in the three *Brassica* species might play important roles in the response of salt stress. In addition, *CPP* genes might be involved in the regulation of salt stress related gene expression through positive or negative regulation, and the former might be dominant. By comparing the expression changes of three homoeologous gene pairs with potential direct evolutionary relationship, we found that the expression trend of *CPP* genes in *B. napus* was consistent with that in the progenitors, but the degree of change was different. It was speculated that the function of *CPP* genes in response to salt stress might be well preserved after allopolyploidization, and the change in response degree might help *BnCPP* genes to respond better and faster to salt stress. Our study can provide reference for the study of molecular resistance mechanism of *B. napus*.

### The Physiological Response of *B. napus* to Salt Stress Showed Its Resistance Advantage

Polyploids may have better stress resistance than diploids, and studies have shown that polyploids are more capable of producing beneficial substances and reducing harmful substances than diploids when subjected to abiotic stress. For example, more proline and less malondialdehyde accumulate in tetraploid rice than diploid rice under salt stress, and polyploid beet had lower Na^+^/K^+^ ratio than diploid beet in response to salt stress (Tu et al., 2014; Zhu, 2016; Wu et al., 2019). When plants are in an unfavorable environment, reactive oxygen species (ROS; such as O_2_, H_2_O_2_, OH^–^) accumulate in cells, which gradually damage cells and plants. In order to reduce this damage, plants have built a set of enzymes to maintain the balance of reactive oxygen species in cells. These enzymes include SOD, POD, and CAT. In this study, we systematically studied the physiological responses of *B. napus* and its progenitors to salt stress, including the activity of SOD, POD, and CAT, and the content of soluble protein, soluble sugar, proline, and chlorophyll. Plants can help them adapt to stress by scavenging ROS accumulated inside cells when they are stressed. Therefore, the increased activities of SOD, POD, and CAT may be related to the response of plants to stress. Proline can be used as an osmotic regulator and hydroxyl radical scavenging agent to help stabilize the subcellular structure of plants, and the accumulation of proline contributes to the resistance of plants (Lv et al., 2011). In addition, plants produce a series of proteins related to stress resistance to avoid injury, and soluble proteins can help plants adapt to adversity as functional proteins or maintainers of osmotic potential (Wu et al., 2004). The increase of soluble sugar content is also a strategy for plants to adapt to adversity, as it not only acts as an osmotic regulator, but also provides carbon framework and energy for the synthesis of other organic solutes (Parankusam et al., 2017). As the basis for material and energy exchange with the outside world, chlorophyll content in plant leaves also changes under stress. The decrease of chlorophyll content was observed in *B. rapa* after salt stress in this study, which is consistent with most of the previous studies, however, the opposite phenomenon was observed in *B. oleracea* and *B. napus*, and the reason may be that the salt stress in our study does not cause rapid decomposition of chlorophyll in the leaves, and this phenomenon may also be related to chloroplast shrinkage (Chaves et al., 2009). In addition, compared with the two diploid progenitors, SOD activity and the accumulation of three osmotic regulatory substances were all the highest in *B. napus* before salt stress, and SOD activity was still relatively high and the accumulation of three osmotic regulatory substances was still the highest in *B. napus* after salt stress. Moreover, POD and CAT activities were inhibited and chlorophyll content decreased in *B. rapa*, while POD and CAT activities and chlorophyll content increased in *B. napus* under salt stress. These results suggest that not all physiological response pathways in allotetraploid *B. napus* were superior to their diploid progenitors *B. rapa* and *B. oleracea* in salt stress, but overall, *B. napus* showed a stronger and more stable physiological response in this study.

## Conclusion

The CPP gene family in *B. napus* expanded after allopolyploidization, and the genome doubling and transposon mediated gene replication might be the main reasons for the expansion. All identified CPP members can be clearly divided into two clades (I, II), and the gene structure of *CPP* in clade I was more conserved than that in clade II. The intron increasing phenomenon was observed in some *CPP* genes after allopolyploidization. Although the gene structure of some *CPP* genes were not changed, the expression patterns of them were changed in *B. napus* after allopolyploidization. Some molecular basis might be associated with the adaptability of *B. napus*, including the expansion of the CPP gene family, the acquisition of introns by some *BnCPPs*, and abundant *cis*-acting elements upstream of *BnCPPs*. A total of 11 *CPP* genes potentially involved in the response to salt stress, and the response degree of some *CPP* genes to salt stress might change in *B. napus* after allopolyploidization. The physiological response of salt stress was stronger and more stable in *B. napus* than its two progenitors. Our study provides critical reference for exploring the potential advantageous molecular basis of various stress response in allopolyploids.

## Data Availability Statement

The original contributions presented in the study are included in the article/Supplementary Material, further inquiries can be directed to the corresponding author/s.

## Author Contributions

ML conceived and drafted the manuscript, performed the data analysis, software analysis, and correction. FW performed the software analysis, surveyed the literature, validated experiments, and visualized the results. JM conducted software analysis, data analysis, and edited and revised the writing. HL performed the software analysis, surveyed the literature, and visualized the results. HY provided the methodology, performed the software analysis, and edited and revised the writing. PZ provided the methodology, guided the writing and editing, and prodded them. JW conceived the manuscript, did project management and data management, directed writing and editing, and provided funding. All authors contributed to the article and approved the submitted version.

## Conflict of Interest

The authors declare that the research was conducted in the absence of any commercial or financial relationships that could be construed as a potential conflict of interest.

## Publisher’s Note

All claims expressed in this article are solely those of the authors and do not necessarily represent those of their affiliated organizations, or those of the publisher, the editors and the reviewers. Any product that may be evaluated in this article, or claim that may be made by its manufacturer, is not guaranteed or endorsed by the publisher.

## Footnotes

1
https://www.Arabidopsis.org/

2
http://pfam.xfam.org/

3
http://smart.embl-heidelberg.de/

4
http://www.ncbi.nlm.nih.gov/Structure/cdd/wrpsb.cgi/

5
http://pdgd.njau.edu.cn:8080/

6
http://itol.embl.de/

7
http://meme-suite.org/

8
http://gsds.cbi.pku.edu.cn/

9
https://gaow.github.io/genetic-analysis-software/m/mapinspect/

10
https://mapinspect.software.informer.com/download/

11
https://web.expasy.org/tools/

12
https://wolfpsort.hgc.jp/

13
http://bioinformatics.psb.ugent.be/webtools/plantcare/html/

## Supplementary Material

Supplementary Figure 1 Phenotype figure of *B. rapa*, *B. napus*, and *B. oleracea* under salt stress.

Supplementary Figure 2 Bar chart of the number of genes for five duplication modes.

Supplementary Figure 3 Visual display of segmental duplicated gene pairs and five duplication modes. Blue lines, red lines, and gray lines represented the segmental duplicated gene pairs in *B. rapa*, *B. napus*, and *B. oleracea*, respectively. Red, yellow, green, blue, and pink circular markers represented WGD, TRD, TD, PD, and DSD, respectively.

Supplementary Figure 4 The CXC domain location of CPP proteins in clade I **(A)** and clade II **(B)** from *B. napus* and its diploid progenitors.

Supplementary Figure 5 Alignment of the conserved CXC domain sequences of CPP proteins from *B. napus* and its diploid progenitors.

Supplementary Figure 6 Conserved motif characterization of CPP proteins in clade I (A) and clade II **(B)** from *B. napus* and diploid ancestors. The red circle indicates orthologs with potential direct evolutionary relationship.
