# Supplementary figures and images for: Comprehensive Evolutionary Analysis of CPP Genes in Brassica napus L. and Its Two Diploid Progenitors Revealing the Potential Molecular Basis of Allopolyploid Adaptive Advantage Under Salt Stress
Source: Front Plant Sci. 2022 Apr 25;13:873071. doi: 10.3389/fpls.2022.873071 (PMC9085292; doi:10.3389/fpls.2022.873071)

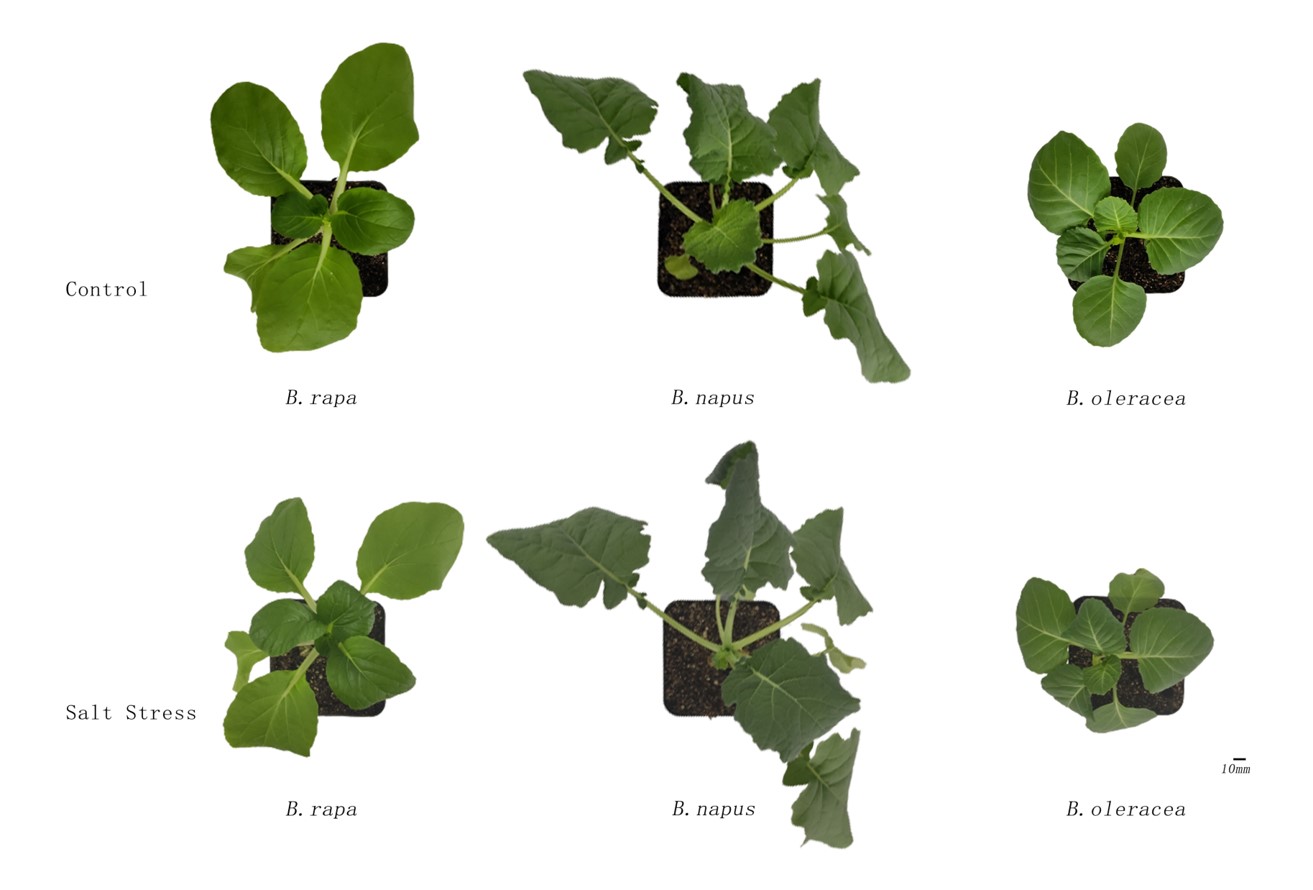

Supplement: Supplementary Figure 1 — Phenotype figure of B. rapa, B. napus, and B. oleracea under salt stress. [file Image_1.JPEG]

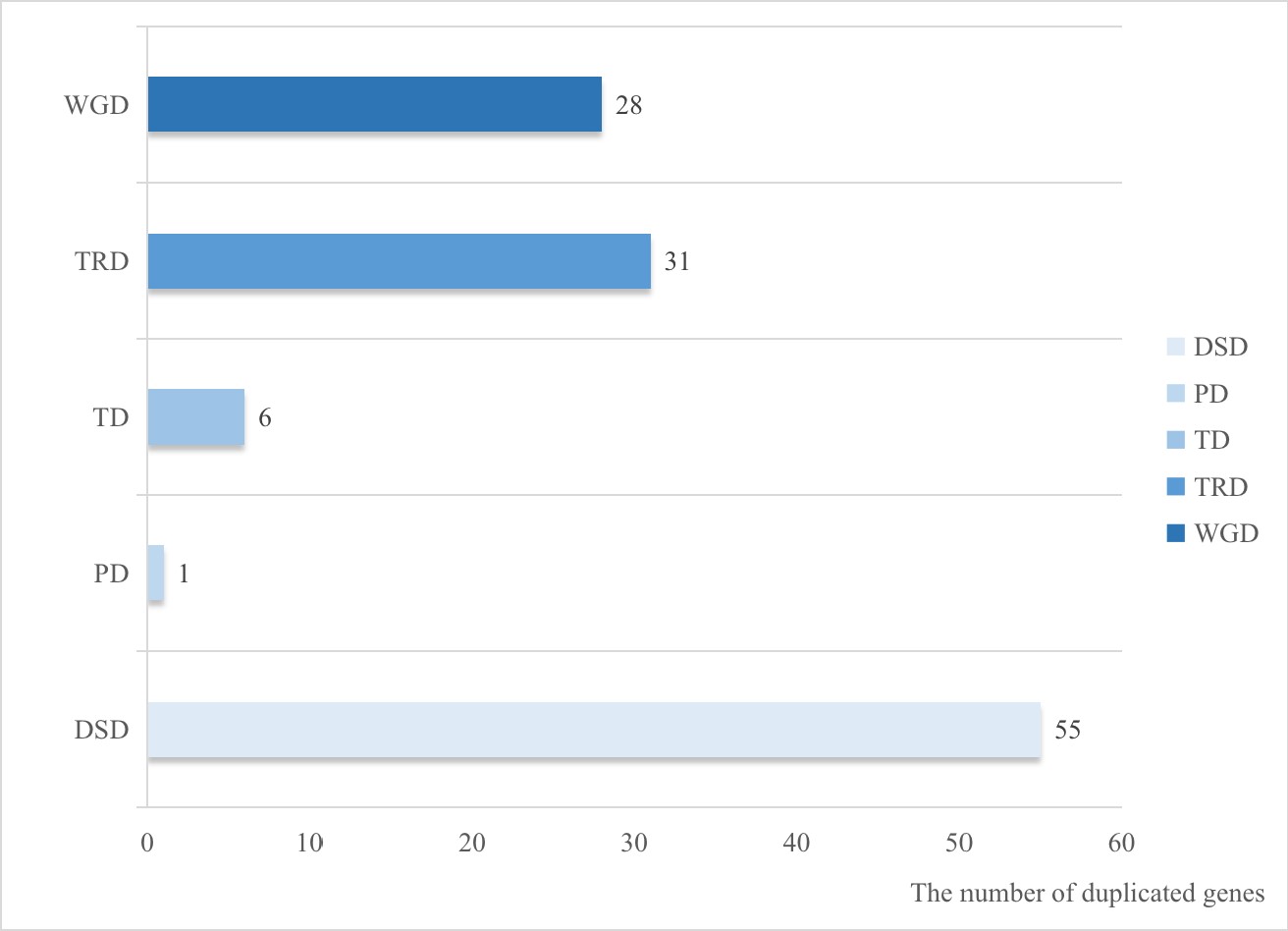

Supplement: Supplementary Figure 2 — Bar chart of the number of genes for five duplication modes. [file Image_2.JPEG]

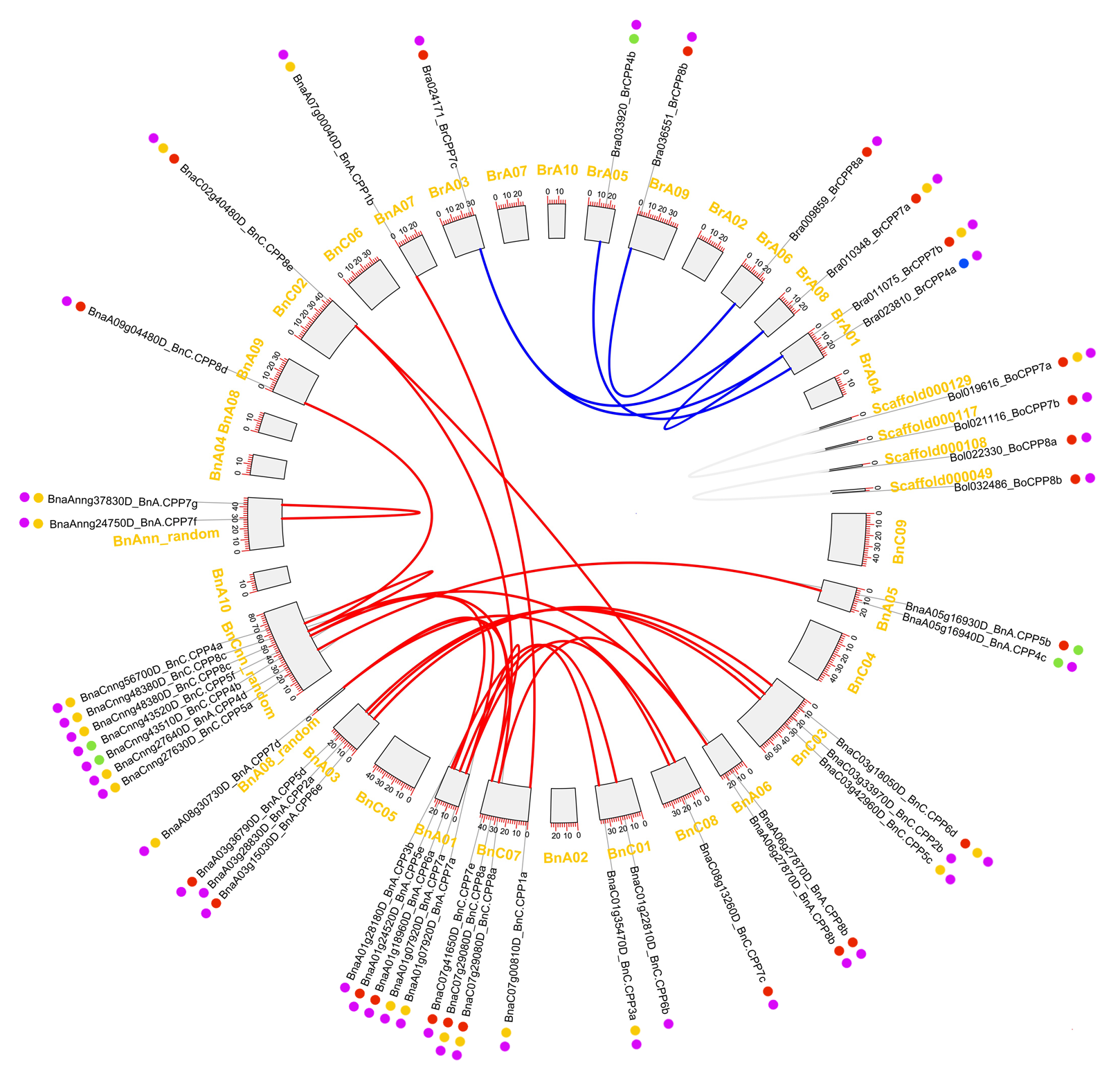

Supplement: Supplementary Figure 3 — Visual display of segmental duplicated gene pairs and five duplication modes. Blue lines, red lines, and gray lines represented the segmental duplicated gene pairs in B. rapa, B. napus, and B. oleracea, respectively. Red, yellow, green, blue, and pink circular markers represented WGD, TRD, TD, PD, and DSD, respectively. [file Image_3.TIF]

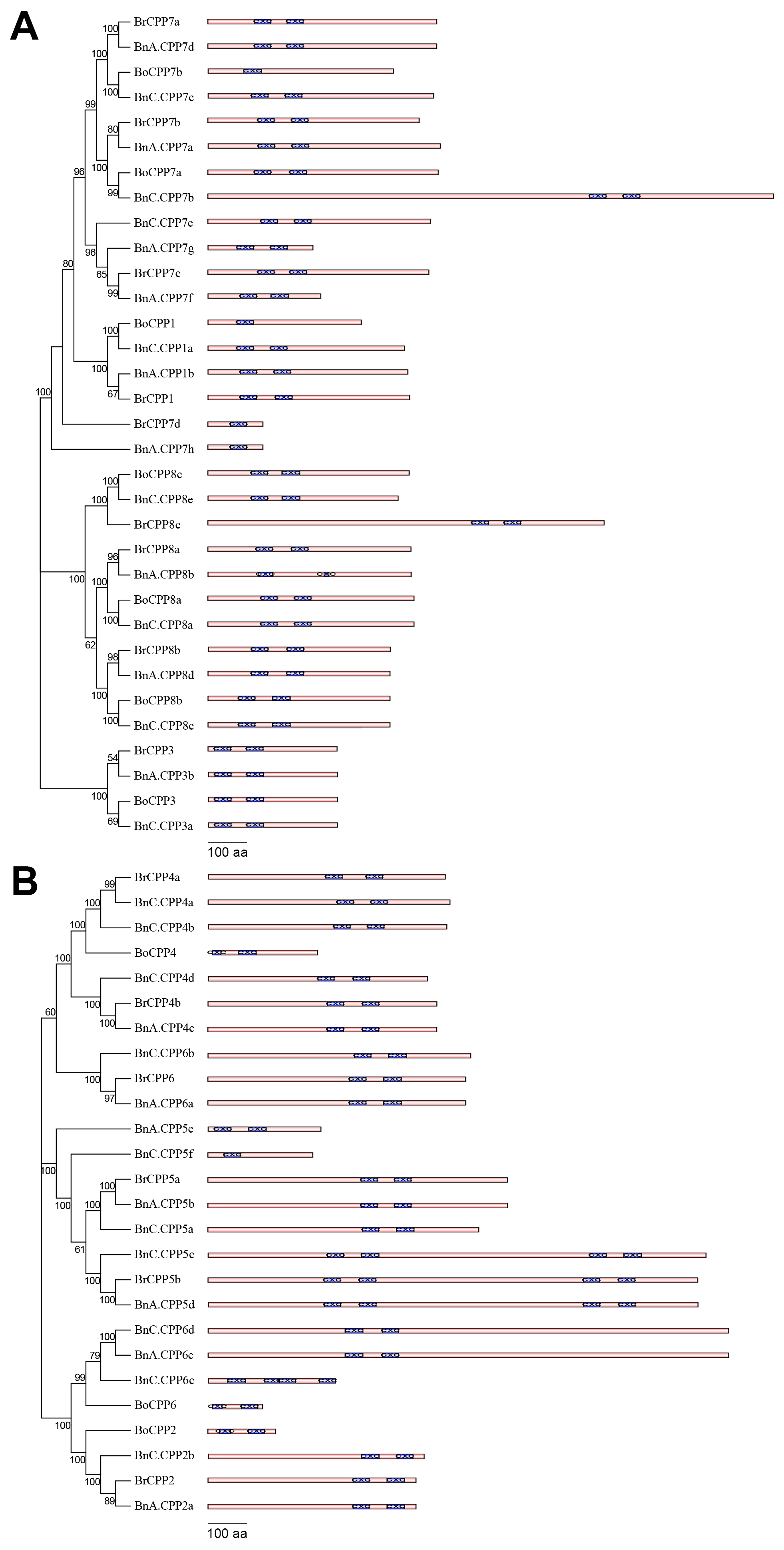

Supplement: Supplementary Figure 4 — The CXC domain location of CPP proteins in clade I (A) and clade II (B) from B. napus and its diploid progenitors. [file Image_4.TIF]

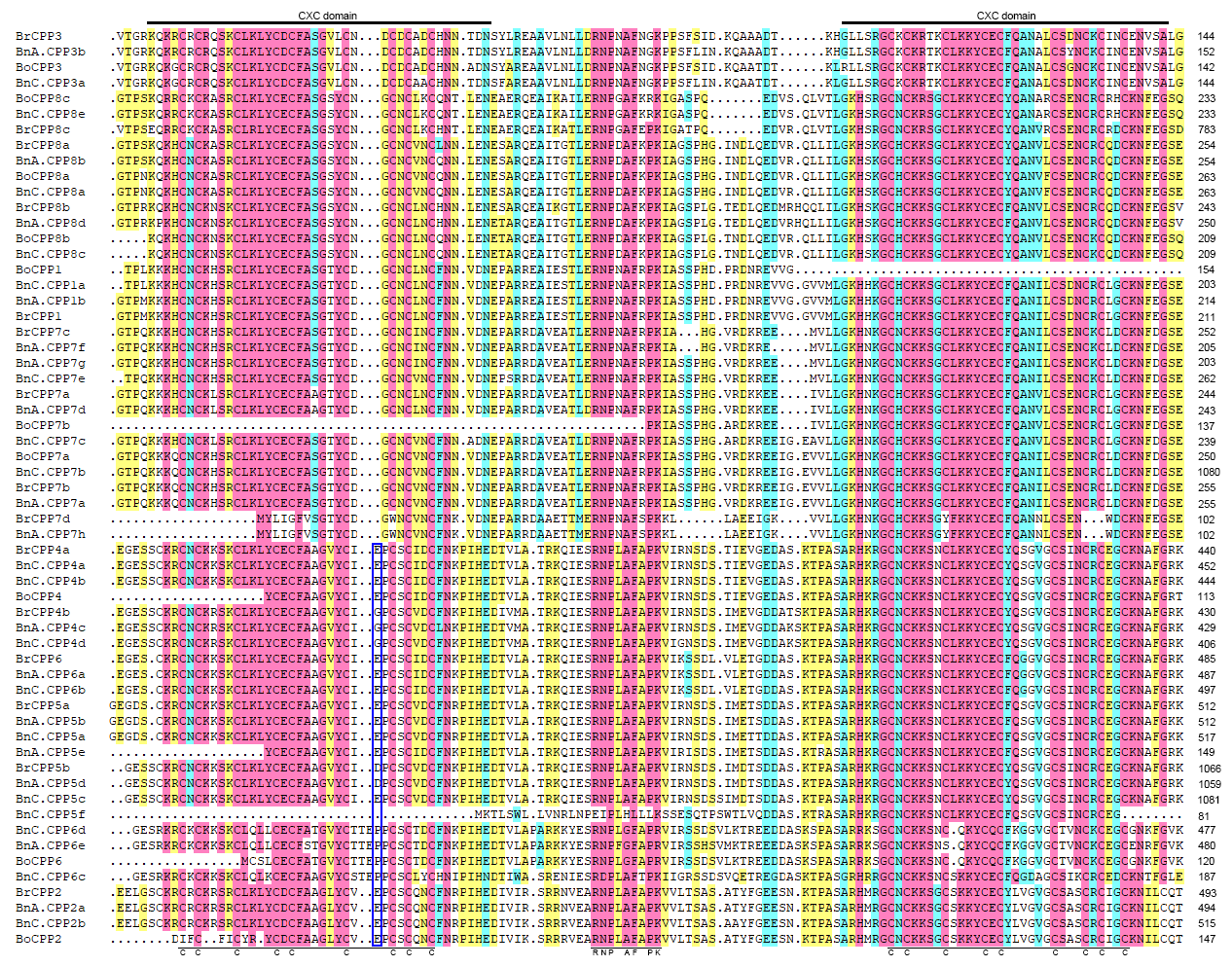

Supplement: Supplementary Figure 5 — Alignment of the conserved CXC domain sequences of CPP proteins from B. napus and its diploid progenitors. [file Image_5.TIF]

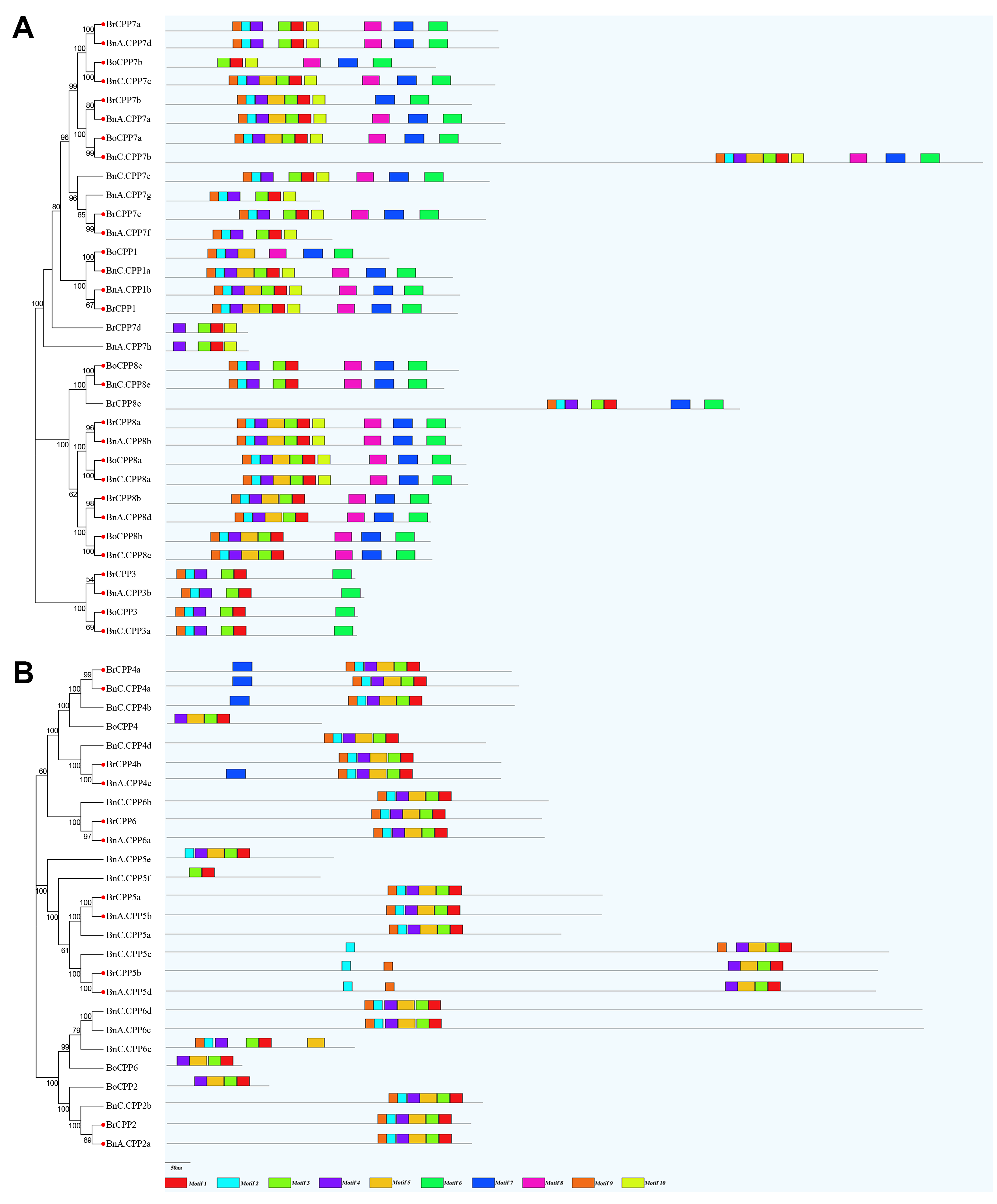

Supplement: Supplementary Figure 6 — Conserved motif characterization of CPP proteins in clade I (A) and clade II (B) from B. napus and diploid ancestors. The red circle indicates orthologs with potential direct evolutionary relationship. [file Image_6.TIF]
