# Supplementary material for: Comprehensive Evolutionary Analysis of CPP Genes in Brassica napus L. and Its Two Diploid Progenitors Revealing the Potential Molecular Basis of Allopolyploid Adaptive Advantage Under Salt Stress
Source: Front Plant Sci. 2022 Apr 25;13:873071. doi: 10.3389/fpls.2022.873071 (PMC9085292; doi:10.3389/fpls.2022.873071)
Supplement: Supplementary file 7 [file Table_1.DOCX]

**TABLE S1. Primers for the qRT-PCR experiment.**

| Gene name | Forward or reverse primer | Primer Sequence |
| --- | --- | --- |
| *BrCPP1* | Forward | GGAGGCCCATCCAAAGTTGTATA |
| *BrCPP1* | Reverse | GACAGTGACTTTCCTTTTGAGCC |
| *BrCPP4a* | Forward | CAATACCACTTCCACCTTCAACAC |
| *BrCPP4a* | Reverse | CCCTTTTGCTGTTTGGAGATAAGG |
| *BrCPP8c* | Forward | TGACATGGGTTCTTGGAGAAGG |
| *BrCPP8c* | Reverse | ATCTCATCAACTGCACAGCTCA |
| *BoCPP1* | Forward | CTGTGAAAGTAGAGCAGGTGGT |
| *BoCPP1* | Reverse | TTGAGGCTGTTGAGTTTGTGTG |
| *BoCPP2* | Forward | CTGGTCTATACTGTGTCGAGCC |
| *BoCPP2* | Reverse | CTAATGGGTTGCGAGCTTCAAC |
| *BoCPP4* | Forward | CGGAACAACAATACCACTTCCAC |
| *BoCPP4* | Reverse | TGATGTTGGTTACTGGAGAGTGG |
| *BoCPP8c* | Forward | CGATCAAACAGTCCCATCCTGT |
| *BoCPP8c* | Reverse | ATCATCATCGGACTCAGCTTCC |
| *BnA.CPP1b* | Forward | TCCTGGTAGCTGTATCTGAGGAA |
| *BnA.CPP1b* | Reverse | CACCGAACAATTAGACGAAGCTG |
| *BnC.CPP2b* | Forward | GCGCTGCTTGACTTTTGATTTG |
| *BnC.CPP2b* | Reverse | GAGGGGTTCACGAGAGCATTAA |
| *BnC.CPP3a* | Forward | TCTTGATTCTCTCGAGTCCCCT |
| *BnC.CPP3a* | Reverse | TTCCACAATACCGCTCTCAGAC |
| *BnC.CPP4a* | Forward | CCATTGGCTCAACTTCCAATCTC |
| *BnC.CPP4a* | Reverse | GTTTCTTCTCCAAGGAGTCGTCT |
| *BnC.CPP8e* | Forward | CGATCAAACAGTCCCATCCTGT |
| *BnC.CPP8e* | Reverse | ATCATTGGACTCAGCTTCCGTC |
| *Actin2/7* | Forward | TTCAATGTCCCTGCCATGTA |
| *Actin2/7* | Reverse | GAGACGGAGGATAGCGTGAG |
