# Supplementary material for: Comprehensive Evolutionary Analysis of CPP Genes in Brassica napus L. and Its Two Diploid Progenitors Revealing the Potential Molecular Basis of Allopolyploid Adaptive Advantage Under Salt Stress
Source: Front Plant Sci. 2022 Apr 25;13:873071. doi: 10.3389/fpls.2022.873071 (PMC9085292; doi:10.3389/fpls.2022.873071)
Supplement: Supplementary file 8 [file Table_2.DOCX]

**TABLE S2. The number of *CPP* genes in *B. napus* and its progenitors.**

| *CPP* genes | *B. rapa* | *B. oleracea* | *B. napus*^a^ |
| --- | --- | --- | --- |
| *CPP1* | 1 | 1 | 2 (1+1) |
| *CPP2* | 1 | 1 | 2 (1+1) |
| *CPP3* | 1 | 1 | 2 (1+1) |
| *CPP4* | 2 | 1 | 4 (1+3) |
| *CPP5* | 2 | 0 | 6 (3+3) |
| *CPP6* | 1 | 1 | 5 (2+3) |
| *CPP7* | 4 | 2 | 8 (5+3) |
| *CPP8* | 3 | 3 | 5 (2+3) |
| Total number | 15 | 10 | 34 |

^a^Note: The first and second numbers in brackets represent the number of genes on the A_n_ and C_n_ subgenomes of *B. napus*, respectively.
