# Supplementary material for: Comprehensive Evolutionary Analysis of CPP Genes in Brassica napus L. and Its Two Diploid Progenitors Revealing the Potential Molecular Basis of Allopolyploid Adaptive Advantage Under Salt Stress
Source: Front Plant Sci. 2022 Apr 25;13:873071. doi: 10.3389/fpls.2022.873071 (PMC9085292; doi:10.3389/fpls.2022.873071)
Supplement: Supplementary file 9 [file Table_3.DOCX]

**TABLE S3.** **The selection pressure of CPP gene family from *B. napus* and diploid ancestors during evolution.**

| Duplicated gene pairs | Ka | Ks | Ka/Ks | Types of selection |
| --- | --- | --- | --- | --- |
| *BnA.CPP1b-BnC.CPP1a* | 0.0205 | 0.1082 | 0.189463956 | Purify selection |
| *BnA.CPP3b-BnC.CPP3a* | 0.03 | 0.0692 | 0.433526012 | Purify selection |
| *BnA.CPP5d-BnC.CPP5c* | 0.0617 | 0.1439 | 0.428769979 | Purify selection |
| *BnA.CPP6a-BnC.CPP6b* | 0.0327 | 0.0563 | 0.580817052 | Purify selection |
| *BnA.CPP7a-BnC.CPP7c* | 0.0935 | 0.2965 | 0.3153457 | Purify selection |
| *BnA.CPP7a-BnC.CPP7e* | 0.0992 | 0.351 | 0.282621083 | Purify selection |
| *BnA.CPP7d-BnC.CPP7c* | 0.033 | 0.1112 | 0.29676259 | Purify selection |
| *BnA.CPP7d-BnA.CPP7a* | 0.1184 | 0.3242 | 0.365206663 | Purify selection |
| *BnA.CPP7d-BnC.CPP7e* | 0.1198 | 0.3334 | 0.359328134 | Purify selection |
| *BnA.CPP7g-BnA.CPP7f* | 0.0365 | 0.0977 | 0.373592631 | Purify selection |
| *BnA.CPP8d-BnC.CPP8c* | 0.0633 | 0.1304 | 0.485429448 | Purify selection |
| *BnC.CPP2b-BnA.CPP2a* | 0.0612 | 0.1064 | 0.57518797 | Purify selection |
| *BnC.CPP4b-BnC.CPP4a* | 0.037 | 0.1175 | 0.314893617 | Purify selection |
| *BnC.CPP4d-BnA.CPP4c* | 0.0323 | 0.0606 | 0.5330033 | Purify selection |
| *BnC.CPP5a-BnA.CPP5b* | 0.0274 | 0.1133 | 0.241835834 | Purify selection |
| *BnC.CPP5f-BnA.CPP5e* | 0.227 | 0.3723 | 0.609723341 | Purify selection |
| *BnC.CPP6d-BnA.CPP6e* | 0.0264 | 0.0586 | 0.450511945 | Purify selection |
| *BnC.CPP8a-BnA.CPP8b* | 0.0327 | 0.0901 | 0.362930078 | Purify selection |
| *BnC.CPP8c-BnC.CPP8a* | 0.148 | 0.3173 | 0.46643555 | Purify selection |
| *BnC.CPP8c-BnA.CPP8b* | 0.1637 | 0.3248 | 0.504002463 | Purify selection |
| *BnC.CPP8e-BnC.CPP8a* | 0.2138 | 0.4283 | 0.499182816 | Purify selection |
| *BnC.CPP8e-BnA.CPP8b* | 0.2134 | 0.4323 | 0.493638677 | Purify selection |
| *BoCPP7a-BoCPP7b* | 0.1152 | 0.2594 | 0.444101773 | Purify selection |
| *BoCPP8a-BoCPP8b* | 0.1483 | 0.309 | 0.479935275 | Purify selection |
| *BrCPP4a-BrCPP4b* | 0.1735 | 0.4784 | 0.362667224 | Purify selection |
| *BrCPP7a-BrCPP7b* | 0.1536 | 0.3793 | 0.404956499 | Purify selection |
| *BrCPP7a-BrCPP7c* | 0.1868 | 0.4552 | 0.410369069 | Purify selection |
| *BrCPP7b-BrCPP7c* | 0.2373 | 0.5626 | 0.421791681 | Purify selection |
| *BrCPP8a-BrCPP8b* | 0.1706 | 0.4299 | 0.396836474 | Purify selection |
