# Supplementary material for: Comprehensive Evolutionary Analysis of CPP Genes in Brassica napus L. and Its Two Diploid Progenitors Revealing the Potential Molecular Basis of Allopolyploid Adaptive Advantage Under Salt Stress
Source: Front Plant Sci. 2022 Apr 25;13:873071. doi: 10.3389/fpls.2022.873071 (PMC9085292; doi:10.3389/fpls.2022.873071)
Supplement: Supplementary file 10 [file Table_4.DOCX]

**TABLE S4. Synteny analysis of *CPP* genes in *A. thaliana*, *B. rapa*, *B. oleracea* and *B. napus*.**

| *AtCPP* genes | Triplication block | *BrCPP* genes in *B. rapa* | | | *BoCPP* genes in *B. oleracea* | | | *BnA.CPP* genes in *B. napus* | | | *BnC.CPP* genes in *B. napus* | | |
| --- | --- | --- | --- | --- | --- | --- | --- | --- | --- | --- | --- | --- | --- |
|  |  | LF | MF1 | MF2 | LF | MF1 | MF2 | LF | MF1 | MF2 | LF | MF1 | MF2 |
| *AtCPP1* | H | *BrCPP1* |  |  | *BoCPP1* |  |  |  |  |  |  |  |  |
| *AtCPP2* | F |  |  | *BrCPP2* |  |  |  |  |  | *BnA.CPP2a* |  |  | *BnC.CPP2b* |
| *AtCPP3* | F |  | *BrCPP3* |  |  | *BoCPP3* |  |  | *BnA.CPP3b* |  |  | *BnC.CPP3a* |  |
| *AtCPP4/ AtCPP5* | F | *BrCPP5a/ BrCPP4b* | *BrCPP4a* | *BrCPP5b* |  | *BoCPP4* |  |  |  |  |  |  |  |
| *AtCPP6* | T | *BrCPP6* |  |  |  |  |  | *BnA.CPP6a* |  |  | *BnC.CPP6b* |  |  |
| *AtCPP7* | U | *BrCPP7b* | *BrCPP7c* | *BrCPP7a* | *BoCPP7a* |  | *BoCPP7b* | *BnA.CPP7a* |  |  | *BnC.CPP7b* | *BnC.CPP7e* | *BnC.CPP7c* |
| *AtCPP8* | Q | *BrCPP8a* |  | *BrCPP8b* | *BoCPP8a* |  | *BoCPP8b* | *BnA.CPP8b* |  | *BnA.CPP8d* | *BnC.CPP8a* |  |  |

Note: LF、MF1 and MF2 represent least-fraction、medium-fraction and most-fraction subgenome, respectively; Triplication blocks mean ancestral genomic blocks of the ancestral Crucifer karyotype.
