# Supplementary material for: Comprehensive Evolutionary Analysis of CPP Genes in Brassica napus L. and Its Two Diploid Progenitors Revealing the Potential Molecular Basis of Allopolyploid Adaptive Advantage Under Salt Stress
Source: Front Plant Sci. 2022 Apr 25;13:873071. doi: 10.3389/fpls.2022.873071 (PMC9085292; doi:10.3389/fpls.2022.873071)
Supplement: Supplementary file 11 [file Table_5.DOCX]

**TABLE S5. The |log_2_FC| of *CPP* genes in flowers, leaves, siliques and stems.**

| Gene name | \|log_2_FC\| in flowers | \|log_2_FC\| in leaves | \|log_2_FC\| in siliques | \|log_2_FC\| in stems |
| --- | --- | --- | --- | --- |
| *CPP1* | 0.84 | 1.57 | 0.41 | 0.99 |
| *CPP3* | 1.82 | 1.81 | 4.91 | 0 |
| *CPP7* | 2.34 | 0.68 | 1.79 | 6.29 |
| *CPP8* | 4.41 | 2.43 | 1.43 | 2.61 |

Note: The value of |log_2_FC| > 1 means the gene expression was biased to *B. rapa*, otherwise, |log_2_FC| < 1 means it was biased to *B. oleracea*, and |log_2_FC| = 0 means this gene had no obvious bias.
